# Supplementary material for: Improving birth preparedness and complication readiness in rural India through an integrated microfinance and health literacy programme: evidence from a quasi-experimental study
Source: BMJ Open. 2022 Feb 18;12(2):e054318. doi: 10.1136/bmjopen-2021-054318 (PMC8860014; doi:10.1136/bmjopen-2021-054318)
Supplement: Author's manuscript [file bmjopen-2021-054318.draft_revisions.pdf]

BMJ Open is committed to open peer review. As part of this commitment we make the peer review history of every article we publish publicly available.

When an article is published we post the peer reviewers' comments and the authors' responses online. We also post the versions of the paper that were used during peer review. These are the versions that the peer review comments apply to.

The versions of the paper that follow are the versions that were submitted during the peer review process. They are not the versions of record or the final published versions. They should not be cited or distributed as the published version of this manuscript.

BMJ Open is an open access journal and the full, final, typeset and author-corrected version of record of the manuscript is available on our site with no access controls, subscription charges or pay-per-view fees (http://bmjopen.bmj.com).

If you have any questions on BMJ Open's open peer review process please email info.bmjopen@bmj.com

BMJ Open

Improving birth preparedness and complication readiness in rural India through an integrated microfinance and health literacy program: Evidence from a quasi-experimental study

Journal:	*BMJ Open*
Manuscript ID	bmjopen-2021-054318
Article Type:	Original research
Date Submitted by the Author:	08-Jun-2021
Complete List of Authors:	Ahmad, Danish; University of Canberra, Health Research Institute,Faculty of Health; Indian Institute of Public Health Gandhinagar (IIPH-G)<br>Mohanty, Itismita ; University of Canberra, Health Research Institute, Faculty of Health<br>Niyonsenga, Theophile; University of Canberra, Health Research Institute, Faculty of Health
Keywords:	Maternal medicine < OBSTETRICS, PUBLIC HEALTH, International health services < HEALTH SERVICES ADMINISTRATION & MANAGEMENT, EPIDEMIOLOGY, OBSTETRICS

SCHOLARONE™
Manuscripts

BMJ

I, the Submitting Author has the right to grant and does grant on behalf of all authors of the Work (as defined in the below author licence), an exclusive licence and/or a non-exclusive licence for contributions from authors who are: i) UK Crown employees; ii) where BMJ has agreed a CC-BY licence shall apply, and/or iii) in accordance with the terms applicable for US Federal Government officers or employees acting as part of their official duties; on a worldwide, perpetual, irrevocable, royalty-free basis to BMJ Publishing Group Ltd ("BMJ") its licensees and where the relevant Journal is co-owned by BMJ to the co-owners of the Journal, to publish the Work in this journal and any other BMJ products and to exploit all rights, as set out in our licence.

The Submitting Author accepts and understands that any supply made under these terms is made by BMJ to the Submitting Author unless you are acting as an employee on behalf of your employer or a postgraduate student of an affiliated institution which is paying any applicable article publishing charge ("APC") for Open Access articles. Where the Submitting Author wishes to make the Work available on an Open Access basis (and intends to pay the relevant APC), the terms of reuse of such Open Access shall be governed by a Creative Commons licence – details of these licences and which Creative Commons licence will apply to this Work are set out in our licence referred to above.

Other than as permitted in any relevant BMJ Author's Self Archiving Policies, I confirm this Work has not been accepted for publication elsewhere, is not being considered for publication elsewhere and does not duplicate material already published. I confirm all authors consent to publication of this Work and authorise the granting of this licence.

For peer review only

Improving birth preparedness and complication readiness in rural India through an integrated microfinance and health literacy program: Evidence from a quasi-experimental study

Danish Ahmad (PhD)[1,2*], Itismita Mohanty (PhD)[1], Theo Niyonsenga (PhD)[1]

1. Health Research Institute, Faculty of Health, University of Canberra, Canberra, Australia
2. Public Health Foundation of India, and Indian Institute of Public Health Gandhinagar (IIPH-G), Gujarat, India

*Corresponding author

Health Research Institute,
Faculty of Health, University of Canberra,
Canberra, Australia
E-mail: drdanish.research@gmail.com
Word count: 4724

For peer review only

ABSTRACT:

Objective: Recently, a novel community health program — the integrated microfinance and health literacy (IMFHL) program was implemented through microfinance-based women's only self-help groups (SHGs) in India to promote birth preparedness and complication readiness (BPCR) to improve maternal health. The study evaluated the impact of the IMFHL program on BPCR practice by women in one of India's poorest states — Uttar Pradesh — adjusting for the community, household and individual variables. The paper also examined for any diffusion of knowledge of BPCR from SHG members receiving the health literacy intervention to non-members in program villages. **Design**: Cross-sectional study. **Settings:** Secondary survey data from the IMFHL project was used. **Participants:** Survey data was collected from 17,244 women in households with SHG member and non-member households in rural India. **Primary and secondary outcomes:** The program's main and secondary effect was evaluated using multivariable logistic regression to estimate adjusted IMFHL program effects on maternal BPCR practice in their last pregnancy **Results:** Membership in SHGs alone is positively associated with BPCR practice, with 17 per cent higher odds of these women practising BPCR compared to women in villages without the programs. Furthermore, the odds of practising complete BPCR increase to almost fifty per cent when a maternal health literacy component is added to the SHGs. A diffusion effect was found for BPCR practice from SHG members to non-members when the health literacy component was integrated into the SHG model. **Conclusions:** The results suggest that SHG membership exerts a positive impact on planned health behaviour and a diffusion effect of BPCR practice from members to non-members when SHGs are enriched with a health literacy component. The study provides evidence to guide the implementation of community health programs seeking to promote BPCR practise in low resource settings.

For peer review only - http://bmjopen.bmj.com/site/about/guidelines.xhtml

Study Strengths and Limitations.

- This is the first study to evaluate the potential of delivering maternal health literacy for pregnancy complications at scale through women based microfinance groups to rural low-income communities where higher maternal health inequities persist.
- The study's evaluation framework and research question adapted the widely used three delays model and the established social determinants of health framework.
- The evaluation design used a novel methodology to identify the program's main effect and secondary effect, adjusting for a comprehensive list of variables using a multivariate stepwise forward selection process to examine how program-associated outcomes are influenced.
- The study was limited by a lack of randomisation in the selection of the survey blocks during program implementation owing to pragmatic operational considerations in a high-population and low-resource setting.

INTRODUCTION

Improving maternal health is a global priority in the attainment of the Sustainable Development Goals (SDGs). Overall, health system reforms and socio-economic improvements in low resource countries led to an almost fifty per cent reduction in maternal deaths by the end of the Millennium Development Goals (MDGs) in 2015 compared to the 1990s (1–3). However, the rate of decline was inadequate to meet the global MDG target of a 75 per cent reduction in the maternal mortality rate (MMR) (3,4). Reflecting this, more than 295,000 maternal deaths occurred in 2017, mostly due to preventable causes across the maternity period, including hypertensive disorders of pregnancy, severe bleeding, obstructed labour and sepsis (4–6), all conditions with established treatment options. The high number of avoidable deaths stemming

For peer review only - http://bmjopen.bmj.com/site/about/guidelines.xhtml

from these causes reflects the residual gap between the MDG target and available means to achieve it. The SDG has a further stringent target of reducing the MMR to 70 per 100,000 live births by 2030 for all countries(5). However, the current situation has been further challenged by severe health service disruptions due to COVID-19. Achieving the SDGs' maternal health target requires concerted action in select high poverty and low resource countries in Sub-Saharan Africa and South Asia, where almost 99 per cent of global preventable maternal deaths occur (1,7,8). India has the world's second-highest number of maternal deaths, with almost 45,000 maternal deaths annually (9,10). Although India invested substantially in improving health service delivery for its predominantly rural populations, high maternal deaths remain a challenge (6,8). Attaining the SDG target requires specific improvements in India to accelerate maternal mortality decline beyond that observed in the MDG era, and the adoption of novel strategies that bridge health system inadequacies with civil society actions (11,12).

The World Health Organization (WHO) recommends skilled birth attendance and access to timely emergency obstetric care during maternal complications as important interventions to improve maternal and neonatal survival (13). Improving birth preparedness and complication readiness (BPCR) is strategic to reduce maternal mortality through increasing awareness and preparedness around emergency obstetrics care in low resource settings (13). According to the WHO, BPCR broadly comprises the following preventative steps: decide on the place of delivery (home or health facility); identify a facility that could provide emergency care; identify people to accompany the woman to the hospital for delivery; identify people to take care of children at home; save/arrange money for delivery expenses or in case of emergency; make advance arrangements for transportation to go to the facility (13).

For peer review only - http://bmjopen.bmj.com/site/about/guidelines.xhtml

Pregnant women and their households are expected to receive BPCR advice as part of antenatal care services provided by health workers (13). While evidence of the effect of BPCR practice on maternal mortality reduction is mixed, a 2017 systematic review showed that the adoption of BPCR as part of an intervention package could improve care-seeking and encourage timely use of health facilities in case of maternal or neonatal complications (14). A study from an urban slum in India showed that skilled attendance at birth was almost 30 per cent higher among well prepared than less prepared mothers (15). Another study from Uganda showed that BPCR knowledge were itself influenced by prior experience of maternal complications and awareness of maternal danger signs (16). However, despite it's demonstrated benefits, studies have shown that BPCR is challenging to achieve in low resource settings where health system services are lacking, and households have low education and high levels of poverty (2,12,16–20).

While India has prioritised institutional delivery through a national conditional cash transfer scheme incentivising deliveries in a health facility, substantial gaps persist in early identification and care of maternal complications, especially in the antenatal and postnatal period for rural populations (10,21,22). Inadequate referral services and limited emergency obstetric care (EmOC) place women at higher risk of adverse maternal outcomes in rural areas (12,21,22). Health system inadequacies are further compounded by community-related delays in seeking care and reaching health facilities due to financial barriers and lack of birth and complication readiness (21,23–27). Maternal death reviews have shown that high poverty and low development states in the north of India, especially Uttar Pradesh, account for the majority of maternal deaths (8,10,28).

Studies from India and rural Uttar Pradesh (UP) have shown that, despite higher institutional deliveries, deficiencies in antenatal care (ANC) limit the transmission of key maternal health information such as BPCR to pregnant women (22,29). In a rural setting, irrespective of risk,

For peer review only - http://bmjopen.bmj.com/site/about/guidelines.xhtml

where antenatal and referral services are inadequate to ensure monitoring of pregnancies, households' adoption of BPCR assumes greater importance (22,29,30).

Limitations in health systems in low resource settings have given rise to alternative approaches, incorporating community efforts to mobilise, educate and empower women to improve maternal health outcomes. Evidence has also shown that health programs led by the community promote the sharing of new knowledge, and this can expand the reach of community interventions (31–33). However, limited research has been conducted in low resource settings to evaluate whether health information provided through community programs facilitates health promotion and even beyond the primary beneficiaries of the program.

Recently, a promising community-based intervention that builds on the concept of empowering poor rural pregnant women by providing them with health literacy and finance-based assistance through women-only microfinance groups was implemented in rural UP. The integrated microfinance and health literacy (IMFHL) program was built on other community-based programs that have shown an improvement in routine indicators of maternal health (34–37), implemented on the microfinance platform to provide pregnant and recently delivered women with health messages. Information is provided to improve knowledge of dangerous signs related to pregnancy, childbirth and post-delivery complications and to adopt birth preparedness and complication readiness practice in order to reduce delays associated with seeking care. Microfinance-based SHG platforms in UP and other low-development regions of India have previously shown that integrated health literacy and SHG interventions can improve routine maternal health practices such as antenatal care utilisation, increase institutional deliveries and also improve newborn health practises at home, such as cord care and timely breastfeeding (38,39). However, the impact of membership in a SHG-based health program on the practice

For peer review only - http://bmjopen.bmj.com/site/about/guidelines.xhtml

For peer review only

of BPCR among SHG members to reduce maternal mortality has not been evaluated. Also, the potential of the program to diffuse health information from members to non-members in villages where SHGs are established is untested.

This research builds on limited evidence on the effectiveness of membership in microfinance programs integrated with a health literacy component on birth preparedness and complication readiness practice among women in rural areas (38–42). It is hypothesised that women in low-income rural households receiving financial access and additional health information through self-help groups (SHGs) would reduce healthcare-seeking delays during maternal complications.

METHODS

Study aim and design

This study evaluated the impact of membership in an integrated microfinance and health literacy (IMFHL) program on the practice of birth preparedness and complication readiness (BPCR) by women during their last pregnancy in rural UP. It also evaluated whether the IMFHL program contributed to the adoption of BPCR among women who did not participate in the IMFHL program as SHG members through a process of diffusion of behaviours from SHG member to neighbouring non-member households in the same villages where the program was implemented.

The study used data from a quasi-experimental survey design, collected as part of the IMFHL project in two rounds (pre-post program implementation), capturing the program's characteristics and other factors in the first round in 2015, followed by a second round of data collection two years into the program in 2017 (38,39). The IMFHL program was implemented over five years (2012–2017). The maternal and neonatal health literacy component was layered

For peer review only - http://bmjopen.bmj.com/site/about/guidelines.xhtml

upon a microfinance-based SHG platform towards 2015 to provide pregnant and recently delivered women with health messages in rural UP. A detailed description of the IMFHL program context, selection of intervention, comparison and control blocks is available elsewhere (38,39).

A woman was classified as a SHG member if she was a member herself or someone else in the household, usually a mother-in-law or sister-in-law, was a member (38). These SHG members represented the first tier of beneficiaries who directly received health information, encouraging them to adopt desired health behaviours to promote maternal and neonatal health. As members and non-members lived in the same villages, it was expected that members receiving health information would communicate knowledge to neighbouring non-member households through informal communication networks that would gradually lead to the adoption of desired behaviours, as observed elsewhere in previous social network analysis studies (42). The process of this social transfer of health information and practices through informal networking to non-members is known as the diffusion effect, also evaluated in this paper (42). This diffusion of knowledge from members (tier I) to neighbouring non-members (tier II) is depicted in Figure 1 below.

Figure 1: Assumed pathway of knowledge diffusion from member households (tier I) to neighbouring non-member households (tier II) within villages

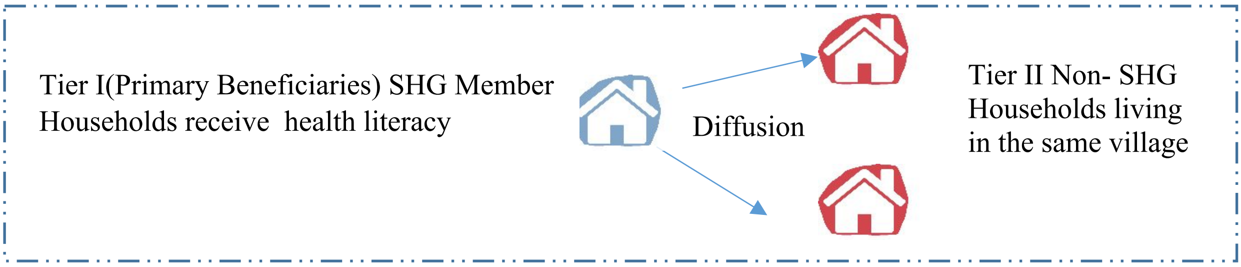

For peer review only - http://bmjopen.bmj.com/site/about/guidelines.xhtml

Study setting and participants

Sampling procedure

The survey sampling design followed UP's administrative hierarchy and collected data from households in *gram panchayats* (GPs) or villages from 70 blocks in 20 districts, aiming for a representative sample from the program's coverage area (43). Moreover, while the same GPs, blocks and districts were visited in both survey rounds, different households were sampled and interviewed in each round. A detailed description of the selection strategy is available elsewhere (38,39).

Sample size and data

The analytical sample used in this study comprised a total of 17,244 eligible women, of whom 59 per cent (10,097 women) did not belong to SHGs. The data was collected at the individual, household and community levels using separate structured questionnaires. The survey collected data from currently married women aged 15 to 49 years who had delivered an infant in the 12 months preceding the survey, and from the household head and village representatives (38, 39). The sample size determination details are reported elsewhere (38,39)

Statistical analysis

A multivariable logistic regression modelling approach was used to evaluate the intervention program's impact on the level of birth preparedness and complication readiness practice among women in rural UP, adjusting for various confounding variables. The main explanatory variable, IMFHL intervention, was categorised according to four levels of household exposure to the IMFHL program, as briefly outlined below. Four separate regression models were fitted to the data, with the first model (Model I) establishing the program's main effect without any confounding variables but including the survey round variable. The second model (Model II)

For peer review only - http://bmjopen.bmj.com/site/about/guidelines.xhtml

included an interaction term (IMFHL intervention by survey round) to draw out the change over time in the effect of IMFHL program exposure. Confounders related to individual health were included in Model III, while the full model, Model IV, included socio-demographic, economic and area-level variables.

Outcome, exposure and confounding variables

Outcome variable:

The birth preparedness and complication readiness (BPCR) practice captured eligible women's self-reported BPCR steps taken during the last pregnancy. The outcome variable, a binary variable, was constructed with '0' representing partial or no BPCR preparation and '1' representing complete BPCR, defined as households that practised all birth preparedness and complication readiness steps during the last pregnancy as outlined by the WHO. In both survey rounds, eligible women, independent of the place of delivery, were asked to recall multiple responses to the question: "*What advance preparation did you/your family members make to manage in case of any pregnancy/delivery complications?*". The interview responses were marked against eight key steps required to achieve BPCR such as: (1) 'Decided on the place of delivery—home or health facility'; (2) 'Knew the facility that could provide emergency care'; (3) 'Identified institution where to rush in case of emergency'; (4) 'Identified people to accompany the woman', (5) 'Identified people to take care of children at home'; (6) 'Saved/arranged money for delivery expense or in case of emergency'; (7) 'Advance arrangement of transportation to go to the facility' and (8) 'others—cloth, soap'.

Main exposure variable.

The exposure variable, the IMFHL intervention, comprised four levels based on households' exposure to the IMFHL program. An ordinal variable was created allowing us to evaluate the

For peer review only - http://bmjopen.bmj.com/site/about/guidelines.xhtml

program's main effect on the practice of BPCR, that is, the magnitude of change in BPCR practice across levels of IMFHL program exposure: intervention (SHG plus health), comparison (SHG only) and pure control (no SHG, no health) households. The coding of the IMFHL explanatory variable, with a description of each group, is shown below:

- **Group 0:** households that were not SHG members (non-members) and were in villages without *any* program intervention (pure control households).
- **Group 1**: households that were not SHG members (non-members) but were in *program villages* where either the SHG program alone or SHG program plus the health intervention was implemented (diffusion-control households).
- **Group 2**: households that were SHG members in program villages where only the SHG program was implemented (comparison households).
- **Group 3**: households that were SHG members and were in villages where both the SHG program and the additional health intervention was provided. Only these households received health literacy intervention through the SHG (intervention households).

Additionally, a survey round variable was created to assess the effect of the intervention program over time (change in women's BPCR in round II in 2017 compared to round I in 2015, when the program was yet to be implemented).

Confounding variables:

A comprehensive set of confounding variables capturing individual, household and community-level characteristics were identified from previous maternal and child health literature and included in models III and IV. The confounding variables in model III represented eligible women's maternal health status and broader maternal health service utilisation indicators. Additional socio-demographic and area-level characteristics were included in the final Model IV. Model IV also included a household wealth quintile variable, constructed for this analysis using Polychoric Principal Component Analysis (PCA), combining all household assets and amenities to evaluate BPCR association across five wealth gradients extending from marginally

For peer review only - http://bmjopen.bmj.com/site/about/guidelines.xhtml

poor (reference category) to the poorest households. Covariates also included a variable to capture household poverty using the income threshold of 'Below Poverty Line card(BPL)' set by the Indian Government.

Descriptive Statistics

Table 1 compiles the levels and steps of birth preparedness and complication readiness (BPCR) practice in the last pregnancy among women aged 15–49 in rural UP (n=17,244). As Table 1 reveals, only 24 per cent of all women in this analysis had not practised any steps for birth or complication readiness, while 49 per cent have undertaken full BPCR in their last pregnancy. A further 6 per cent of women had only taken steps to address birth preparedness, while 21 per cent of all women had only undertaken the three steps required for complication readiness.

For peer review only

Table 1: Levels of BPCR practice in last pregnancy among women aged 15–49 in rural Uttar Pradesh

Level of preparedness	Number (%) (n=17,244)	Specific activity done
No BPCR preparedness	4,187 (24)	
Only birth preparedness done	955 (6)	0. Decided on the place of delivery (at home or health facility)<br>1. Knew the facility that could provide emergency care<br>2. Identified people to accompany the woman<br>3. Identified people to take care of children at home<br>4. Others (oil/cloth/soap)
Only complication readiness done	3,697 (21)	1. Saved/arranged money for delivery expense or in case of emergency<br>2. Advance arrangement of transportation to go to the facility<br>3. Identified institution where to rush in case of emergency
Both birth preparation and complication readiness steps done	8,405 (49)	1. Decided on the place of delivery (at home or health facility)<br>2. Knew the facility that could provide emergency care<br>3. Identified people to accompany the woman

For peer review only - http://bmjopen.bmj.com/site/about/guidelines.xhtml

Level of preparedness	Number (%) (n=17,244)	Specific activity done
		4. Identified people to take care of children at home
		5. Saved/arranged money for delivery expense or in case of emergency
		6. Advance arrangement of transportation to go to the facility
		7. Identified institution where to rush in case of emergency
		8. Others (oil/cloth/soap)
**Total**	**17,244 (100)**	

Notes - BPCR: birth preparedness and complication readiness.

Table 2 presents the descriptive statistics for eligible women categorised across SHG member and non-member households across all variables used in this paper. The study sample included more women from non-member households (59 per cent) than from member households, but with an equal allocation of households within various levels of IMFHL program exposure.

Sampled women had a mean parity of 2.4 (median 2, range 0–9) which reflects the fertility rate in rural UP, but is higher than the current Indian fertility rate (median = 2.2, range: 1–4) (43). Moreover, close to 48 per cent of all surveyed women reported experiencing a pregnancy complication in their last pregnancy, while only a quarter (25%) had suffered a spontaneous or induced abortion. Furthermore, women on average had four contacts with frontline workers in their last pregnancy, with 26 per cent of women receiving four ANC visits with all tests during their last pregnancy and only nine per cent of women receiving the vital three postnatal visits.

Table 2: Summary statistics of variables by non-member households and SHG households

		Summary statistics (N=17,244)	
	**Variable**	**Non-member households**	**SHG households**
	Independent variables: Program exposure characteristics		
1.	**Level of household (HH) microfinance (MF) exposure**	10,097 (59%)	7,147 (41%)
	0. HH in a village with **no SHG, no health** intervention (pure control – reference)	3709 (37%)	---

For peer review only - http://bmjopen.bmj.com/site/about/guidelines.xhtml

For peer review only

	Variable	Summary statistics (N=17,244)	
		Non-member households	SHG households
	1. **Non-member** HH in a village with **SHG only program**	3042 (30%)	---
	2. **Non-member** HH in a village with **SHG plus health** intervention	3346 (33%)	---
	3. **SHG member** HH in a village with **SHG only intervention**	---	3623 (51%)
	4. **SHG plus health member** HH in a village with **SHG *plus* health** intervention	---	3524 (49%)
2.	**Evaluation survey round**		
	Round 1/ baseline-2015 (=0)	5,454 (54%)	3,269 (45%)
	Round 2/ endline-2017 (=1)	4,643 (45%)	3,878 (54%)
	Independent variables: Individual health and health system characteristics		
3.	**Parity (number of previous pregnancies) of the eligible woman (EW)**	Mean=2.4 (SD=1.44)	Mean=2.4 (SD=1.41)
4.	**Any past pregnancy loss (due to spontaneous/induced abortion)**		
	No previous pregnancy loss (reference)	7,547 (75%)	5,257 (74%)
	Previous pregnancy loss	2,550(25%)	1,890(26%)
5.	**Any complication experienced in last pregnancy/labour or post-partum**		
	No complication experienced (reference)	5,313 (53%)	3,710 (52%)
	Complication experienced	4,784 (47%)	3,437 (48%)
6.	**EW's knowledge of the minimum number of antenatal check-ups (ANC) required during pregnancy**		
	Incorrect knowledge (reference)	6,381 (63%)	4,386 (61%)
	Correct knowledge	3,716 (37%)	2,761 (39%)
7.	**Received four or more ANC in last pregnancy (with urine/blood pressure /weight/abdominal/ultrasound tested in last ANC)**		
	Not received (reference)	7,429 (74%)	5,070 (71%)
	Received four ANC with all tests done in last ANC	2,668 (26%)	2,077 (29%)
8.	**Place of last delivery**		
	Home delivery (reference)	1,740 (17%)	1,199 (17%)
	Institutional delivery	8,357 (83%)	5948 (83%)

For peer review only - http://bmjopen.bmj.com/site/about/guidelines.xhtml

	Variable	Summary statistics (N=17,244)	
		Non-member households	SHG households
9.	**Duration (in hours) of stay in health facility immediately after delivery**		
	1. Home delivery (reference)	1,740 (17%)	1,199 (17%)
	2. Discharged within 12 hrs	5,603 (56%)	4,127 (58%)
	3. Discharged between 12–24 hrs	1,134 (11%)	795 (11%)
	4. Discharged between 24–48 hrs	720 (7%)	506 (7%)
	5. Discharged between 48–72 hrs	283 (3%)	146 (2%)
	6. Discharged after >72 hrs	617 (6%)	374 (5%)
10.	**Received three postnatal care visits (PNC) in the first seven days after delivery**		
	Not received	9,188 (91%)	6,488 (91%)
	Received three PNC in first seven days after delivery	909 (9%)	659 (9%)
11.	**Number of contacts with ASHA/ANM/AWW/SHG in last pregnancy**	Mean=4.0 (SD=5.4)	Mean=4.23 (SD=5.4)
12.	**Distance (kilometres) to primary health centre if not available in the village**	Mean=5.4 (SD=4.9)	Mean=5.4 (SD=4.7)
	Independent variables: Socio-demographic/economic and area level characteristics		
13.	**Village distance (kilometres) to closest town**	Mean=1.47 (SD=0.77)	Mean=1.49 (SD=0.75)
14.	**Population of village**	Mean=5,153 (SD=5,134)	Mean=5,140 (SD=5,113)
15.	**Household (HH) with Below Poverty Line (BPL) Card**		
	HH without BPL Card (reference)	5,598 (55%)	3,831(54%)
	HH with BPL Card	4,499 (45%)	3316(46%)
16.	**Household wealth quintile (poor to poorest)**		
	1. Marginally Poor	1,985 (20%)	1,498 (21%)
	2. Moderately Poor	2,049 (20%)	1,502 (21%)
	3. Poor	1,994 (20%)	1,471 (20%)
	4. Poorer	2,029 (20%)	1,396 (20%)
	5. Poorest	2,040 (20%)	1,280 (18%)
17.	**EW Presently Working to earn cash, in kind or both**		
	Not working	8,436 (84%)	5,936 (83%)
	Presently working	1,661 (16%)	1,211 (17%)
18.	**Family type**		
	Nuclear (reference)	4,278 (42%)	2,888 (40%)
	Joint and extended family	5,819 (58%)	4,259 (60%)
19.	**Religion**		
	Muslim (reference)	816 (8%)	601 (8%)
	Hinduism and others	9,281 (92%)	6,546 (92%)
20.	**Scheduled caste**		
	General caste	1,250 (12%)	873 (12%)

For peer review only

For peer review only - http://bmjopen.bmj.com/site/about/guidelines.xhtml

	Variable	Summary statistics (N=17,244)	
		Non-member households	SHG households
	Other backward caste	4,362 (43%)	3,072 (43%)
	Scheduled caste and scheduled tribe	4,485 (45%)	3,202 (45%)
21.	**EW age in completed years**	Mean=25 (SD=4.56)	Mean=25 (SD=4.55)
22.	**EW's education level**		
	No schooling (reference)	3,417 (34%)	2,349 (33%)
	Completed primary/middle school (up to Year 9) and above	6,680 (66%)	4,798 (67%)
23.	**EW's husband education level**		
	No schooling (reference)	1,732 (17%)	1,211 (17%)
	Completed primary/middle school (up to Year 9) and above	8,365 (83%)	5,936 (83%)
	Dependent variable		
24.	**EW's level of birth preparedness and complication readiness (BPCR) practice in last pregnancy**	10,097 (59%)	7,147 (41%)
	Incomplete or no BPCR steps (reference)	5,435 (53%)	3,404 (48%)
	Complete BPCR steps practised in last pregnancy	4,662 (46%)	3,743 (52%)

Acronyms: Eligible woman (EW), Self-help group (SHG), Accredited Social Health Worker (ASHA)/Auxiliary Nurse Midwife (ANM) Anganwadi worker (AWW). ASHA/ANM & AWW are government health workers in villages as per population guidelines providing preventative maternal, child and other health services.

Note: Non-member households are those which do not include a SHG member and SHG households are those that include a SHG member.

Patient and public involvement

This study accessed data collected by the IMFHL project as part of the project evaluation. Neither patients nor the public was involved in the planning, design conduct or reporting of this study.

RESULTS

The results from multivariable logistic regression models are presented in Table 3 for Models I and II (effects of IMFHL program exposure and survey round), and in Table 4 for Models III and IV (effects of IMFHL program exposure and survey round adjusting for socio-

For peer review only

demographic, economic and area-level confounders). The Odds Ratio (OR) along with their associated 95 per cent confidence intervals (95% CI) are reported. The a priori level of significance was set at the usual 5% alpha and all p-values reported in Tables using the asterisk convention: ***: p < 0.01; **: p < 0.05; *: p < 0.10, with the last category meant to show that a 'trend towards statistical significance' has to be noted.

Model I: IMFHL program's effects on birth preparedness and complication readiness

Women belonging to a SHG only and those belonging to and receiving the health intervention from a SHG had higher odds of reporting birth and complication readiness practice in the last pregnancy than non-SHG members in non-program (pure control) villages (Table 3). Women in SHG households (members) were 1.17 times more likely to have practised BPCR in their last pregnancy (OR=1.17, 95% CI: 1.07–1.29, p<0.01) than non-members in pure control villages. SHG members who received additional health literacy were 1.48 times more likely to practise BPCR (OR=1.48, 95% CI: 1.35–1.63, p<0.01) than non-members in pure control villages. In contrast, results also showed that non-member women living in the same villages where either SHG or SHG plus health intervention was implemented were 0.89 times *less likely* to practise BPCR compared to non-member women in pure control villages, that is, women in those villages where no SHG or health intervention was implemented (OR=0.89, 95% CI:0.82–0.97, p<0.01). Overall, results showed that women in households interviewed in round II were almost 0.37 times *less likely* to have practised BPCR compared to households interviewed in round I given all the levels of program exposure (OR=0.37, 95% CI:0.35–0.39, p<0.01).

Model II: Interaction effect of IMFHL program and survey round

Model II showed that the inclusion of the interaction term rendered the women in SHG plus health households in round I with a statistically not significant four per cent lower odds of practising BPCR compared with non-member women in control villages in round I (OR=0.96,

For peer review only - http://bmjopen.bmj.com/site/about/guidelines.xhtml

95%CI: 0.84-1.11, p>0.10). As explained, the magnitude observed here corresponds to what is happening in round I (in Model II), while what was observed in Model I (1.48 times more likely to practise BPCR) corresponds to the magnitude of the exposure all rounds combined. And this is expected since there is no program exposure in Round I.

The results from the two-way interaction in Model II of round by households' microfinance exposure level showed that women in SHGs that received the health literacy intervention were 2.21 times more likely to practise BPCR in round II compared to the same household type in round I (OR=2.21, 95%CI: 1.82–2.68, p<0.01). This means that women in the intervention group were roughly 2.122 (=0.96*2.21) (95%CI:1.52–2.97) times more likely to practise BPCR in round II compared to women in control villages. Similarly, the results showed that non-members in program villages reported 1.73 times higher odds of BPCR practice in round II compared to the same household type in round I (OR=1.73, 95%CI: 1.46–2.05, p<0.01). These results indicate that a diffusion effect occurred from SHG women receiving health literacy to non-member women in the same villages, with women in this group being almost 1.194 (=0.69*1.73) (95%CI:0.89-1.57) times more likely to practise BPCR.

Models III and IV: Effects of IMFHL program adjusted for confounders

Overall, the effects of program exposure on BPCR observed previously in model II remained unaffected when adjusting for confounders in models III and IV, suggesting that the outcome, BPCR, is mainly influenced by program exposure rather than confounders.

Key confounders: Effects of individual health and health system variables

Women who were discharged earlier from the health facility after delivery had higher odds of reporting the practice of all steps of BPCR in their last pregnancy (OR=1.15, 95%CI: 1.00–1.33, p<0.05). In contrast, women who reported receiving four ANC visits with key tests done

For peer review only - http://bmjopen.bmj.com/site/about/guidelines.xhtml

in the last ANC visit reported *lower odds* of BPCR practice compared to women who received less than four visits and with incomplete tests (OR=0.90, 95%CI:0.82–0.99, p<0.05). Among variables reflecting the continuum of maternal care, women with the correct knowledge about the required number of ANC visits were more likely to practise BPCR compared to those with incorrect knowledge (OR=1.06, 95%CI: 0.99–1.13, p<0.10).

Key confounders: Effect of socio-demographic, economic and area-level variables

Model IV results for the wealth quintile variable showed that the likelihood of BPCR practice reduced as the level of household poverty increased from marginally poor to the poorest households, with the poorest households (those in the fifth wealth quintile) being 0.87 times less likely to practise BPCR than women in marginally poor households (those in the first quintile). Maternal education, which has previously been found in the literature to be strongly correlated with routine maternal health behaviours, in contrast, was negatively associated with BPCR practice in this analysis. Mothers who had completed at least primary education were seven per cent less likely to practise BPCR than mothers without any schooling (OR=0.93, 95% CI: 0.87–0.99, p<0.05).

Table 3: Logistic regression models I and II results estimating levels of BPCR: Odds ratios and associated 95% confidence intervals

		**Main effects**	
	**Explanatory variable name**	**Model I**	**Model II**
		Odds ratio (95 % CI)	Odds ratio (95 % CI)
	**Main effects**		
1.	**Level of household (HH) microfinance (MF) exposure**		
	0. HH in a village with **no program** intervention (pure control – reference)	----	----
	1. **Non-member** HH in village with **SHG or SHG plus health intervention**	0.89*** (0.82-0.97)	0.69*** (0.61-0.77)
	2. **SHG member** HH in village with **SHG only intervention**	1.17*** (1.07-1.29)	1.13* (0.98-1.30)
	3. **SHG plus health member** HH in village with **SHG *plus* health** intervention	1.48*** (1.35-1.63)	0.96 (0.84-1.11)
2.	**Round**		

For peer review only - http://bmjopen.bmj.com/site/about/guidelines.xhtml

	Round 1 (reference)		
	Round 2	0.37*** (0.35-0.39)	0.25*** (0.22-0.29)
3.	**Two-way interaction effects: Round # households (HH) microfinance (MF) Exposure**		
	Round 1# Non-MF HH in pure control village (reference)		
	Round 2 # Non-MF HH in a village with MF or MF plus health	----	1.73*** (1.46-2.05)
	Round2 # **SHG member** HH in village with MF program only	----	1.10 (0.90-1.33)
	Round2 # **SHG plus health member** HH in village with MF plus health intervention	----	2.21*** (1.82-2.68)

Note: Confidence intervals in parentheses; significant p-value shown as ***: p<0.01, **: p<0.05 and *: p<0.10. **BPCR:** birth preparedness and complication readiness.

Table 4: Logistic regression models III and IV results estimating levels of BPCR using confounders: Odds ratios and associated 95% confidence intervals

		**Secondary effects using confounders**	
**S.no**	**Explanatory variable name**	**Model III**	**Model IV**
		Odds ratio (95 % CI)	Odds ratio (95 % CI)
1.	**Level of household (HH) microfinance (MF) exposure**		
	1. HH in a village with **no program** intervention (Pure control –reference)		
	2. **Non-member** HH in a village with **SHG or SHG plus health intervention**	0.69*** (0.61-0.78)	0.70*** (0.62-0.78)
	3. **SHG member** HH in a village with **SHG only intervention**	1.13* (0.98-1.30)	1.14* (0.99-1.31)
	4. **SHG plus health member** HH in a village with **SHG *plus* health** intervention	0.97 (0.84-1.11)	0.98 (0.85-1.12)
2	**Round**		
	Round 1 (reference)		
	Round 2	0.24*** (0.21-0.28)	0.24*** (0.20-0.27)
3.	**Two-way interaction effects (round # SHG exposure)**		
	Round 1# Non-MF HH in pure control village (reference)		
	Round 2 # Non-MF HH in village with MF or MF plus health	1.72*** (1.45-2.04)	1.72*** (1.45-2.04)
	Round2 # MF-HH in village with MF only	1.10 (0.90-1.33)	1.10 (0.90-1.33)
	Round2 # MF plus health HH in village with MF plus health	2.20*** (1.82-2.67)	2.20*** (1.81-2.66)
	Model III – Individual health and health system variables		
4.	**Parity (number of previous pregnancies) of Eligible Woman (EW)**	0.98 (0.96-1.00)	0.98 (0.95-1.01)

For peer review only - http://bmjopen.bmj.com/site/about/guidelines.xhtml

		Secondary effects using confounders	
**S.no**	**Explanatory variable name**	**Model III**	**Model IV**
5.	**Any past pregnancy loss (due to spontaneous/induced abortion)**		
	No previous pregnancy loss (reference)		
	Previous pregnancy loss	0.98 (0.91-1.05)	0.98 (0.90-1.05)
6.	**Any complication experienced in last pregnancy/labour or postpartum**		
	No complication experienced (reference)		
	Complication experienced	0.95 (0.89-1.01)	0.95 (0.89-1.01)
7.	**EW's knowledge of the minimum number of antenatal check-ups (ANC) required during pregnancy**		
	Incorrect knowledge (reference)		
	Correct knowledge	1.07** (1.00-1.14)	1.06* (0.99-1.13)
8.	**Received four or more ANC in last pregnancy (with urine/blood pressure /weight/abdominal/ultrasound test in last ANC)**		
	Not received (reference)		
	Received four ANC with all tests done in last ANC	0.92 (0.85-1.01)	0.91** (0.82-0.99)
9.	**Place of last delivery**		
	Home delivery (reference)		
	Institutional delivery	0.95 (0.81-1.11)	0.92 (0.79-1.08)
10.	**Duration (in hours) of stay in health facility immediately after delivery**		
	Home delivery (reference)		
	Discharged within 12 hrs	1.14* (0.98-1.31)	1.15** (0.99-1.33)
	Discharged between 12–24 hrs	1.09 (0.92-1.29)	1.10 (0.93-1.30)
	Discharged between 24–48 hrs	0.98 (0.82-1.17)	0.99 (0.83-1.18)
	Discharged between 48–72 hrs	1.02 (0.81-1.29)	1.05 (0.83-1.32)
	Discharged after >72 hrs	1.00	1.00
11.	**Received three postnatal care visits (PNC) in first seven days after delivery**		
	Not received any PNC or received after seven days		
	Received three PNC in first seven days after delivery	1.06 (0.95-1.19)	1.05 (0.94-1.18)
12.	**Number of contacts with ASHA/ANM/AWW/SHG in last pregnancy**	0.99 (0.99-1.00)	1.00 (0.99-1.00)
13.	**Distance to primary health centre if not available in the village**	0.99 (0.98-1.00)	0.99 (0.99-1.00)
	Model IV-Socio-demographic/economic and area-level variables		

For peer review only - http://bmjopen.bmj.com/site/about/guidelines.xhtml

		Secondary effects using confounders	
**S.no**	**Explanatory variable name**	**Model III**	**Model IV**
14.	**Village distance (kilometres) to closest town**	----	0.99 (1.03-1.13)
15.	**Population of village**	----	1.00 (0.99-1.00)
16.	**Household (HH) with Below Poverty Line (BPL) Card**		
	No BPL Card (reference)		
	Yes – HH has BPL Card	----	1.01 (0.94-1.08)
17.	**Household wealth quintile (poor to poorest)**		
	1. Marginally poor (reference)		
	2. Moderately poor	----	1.11** (1.00-1.22)
	3. Poor	----	1.00 (0.90-1.10)
	4. Poorer	----	0.91* (0.81-1.01)
	5. Poorest	----	0.87** (0.77-0.97)
18.	**EW presently working to earn cash, in-kind or both**		
	Not working (reference)		
	Currently working	----	1.00 (0.92-1.09)
19.	**Family type**		
	Nuclear (reference)		
	Joint and extended family	----	1.05 (0.98-1.13)
20.	**Religion**		
	Muslim (reference)		
	Hinduism & others	----	1.06 (0.94-1.19)
21.	**Scheduled caste**		
	General caste (reference)		
	Other backward castes	----	0.96 (0.87-1.07)
	Scheduled caste and scheduled tribe	----	0.97 (0.87-1.08)
22.	**EW age in completed years**	----	1.00 (0.99-1.01)
23.	**EW education level**		
	No schooling (reference)		
	Completed primary/middle School (up to Year 9) and above	----	0.96 (0.89-1.03)
24.	**EW's husband education level**		
	No schooling (reference)		

For peer review only

		Secondary effects using confounders	
**S.no**	**Explanatory variable name**	**Model III**	**Model IV**
	Completed primary school/middle school (up to Year 9) and above	----	1.06 (0.97-1.17)
	**Estimation of model fit**	**Model III**	**Model IV**
	Log likelihood	-11,354	-11,334
	Number of observations	17,244	17,244
	AIC/BIC	22,750/22,914	22,741/ 23,021

Notes: Confidence intervals in parentheses; significant p-value shown as ***: p<0.01, **: p<0.05 and *: p<0.10. Log-likelihood and AIC/BIC values are also reported.

Acronyms: Eligible woman (EW), self-help group (SHG), birth preparedness and complication readiness (BCPR), Accredited Social Health Worker (ASHA)/Auxiliary Nurse Midwife (ANM) Anganwadi worker (AWW). ASHA/ANM & AWW are government health workers situated in villages as per population guidelines and provide preventative maternal, child and other health services

DISCUSSION

This study makes a significant contribution to the literature on maternal health promotion and implementation by investigating the main effect of IMFHL program membership on birth preparedness and complication readiness (BPCR) practice among poor women who have recently given birth in rural UP. The study provides evidence of the impacts of SHG membership alone and when integrated with health literacy on BPCR practice. This evidence suggests that BPCR practice is potentially improved during maternal complications among SHG members when SHGs were integrated with the health literacy program and supports the claim that microfinance-based women's groups are an effective strategy to provide health literacy to marginalised rural populations enabling behaviour change. No previous study has examined the impact on BPCR in the context of SHG programs alone or in an integrated SHG and health literacy intervention program in India or elsewhere. This is also the first study to find the presence of a diffusion effect of maternal health promotion behaviour — BPCR practice—from SHG members receiving health literacy to non-members in program villages.

For peer review only - http://bmjopen.bmj.com/site/about/guidelines.xhtml

Overall, the results reveal that SHGs promote the adoption of BPCR practice among members and the odds of BPCR adoption almost doubles when a health literacy component is integrated into the model. However, the diffusion effect of BPCR practice is only observed when SHGs have an integrated health literacy component. The study finds the diffusion of BPCR practices among non-members in SHG plus health literacy villages and not in the SHG-only villages.

Therefore, these findings suggest that self-help groups require the addition of a maternal health literacy component to achieve a higher coverage of a behavioural health intervention among members and non-members in villages. The underlying mechanism of diffusion requires additional research to explain pathways; however, insights from previous studies suggest that either SHG members may themselves be promoting practices as 'model adopters' of new behaviour through interpersonal contact, or that SHGs serve as a better platform for facilitating linkages between non-members and community health workers when combined with health literacy intervention (42,44,45). It is, therefore, feasible that when SHG members receive health literacy, they may be acting as change agents to encourage non-members to adopt desired health behaviours.

This study highlights the important role that SHGs play as a catalyst for promoting maternal health behavioural change when layered up with health literacy intervention. The findings are supported by other studies from UP and Bihar, another Indian state with low development indicators, that had shown an increase in routine indicators of maternal health system utilisation (such as four ANC visits and institutional delivery) and home-based neonatal health care practices by 5 to 11 percentage points when health literacy intervention was provided through SHGs compared to membership in SHGs alone (39,46). In this study, the higher likelihood of BPCR practise among women in SHGs plus health intervention households could be a direct

For peer review only - http://bmjopen.bmj.com/site/about/guidelines.xhtml

consequence of SHG membership that provides a platform, which gives women access to needed funds. This access addresses one of the key BPCR steps—saving funds for a maternal emergency that influences care-seeking behaviours in low-income families. High maternal health expenses are seen as a strong factor for pushing vulnerable families at risk of poverty into debt traps that reinforce debt, borrowing, and illness cycles. Therefore, in such settings, the provision of health literacy and financial access together are expected to improve households' care-seeking preferences. Previous studies have also shown that SHGs provide mutual support among members (38,44). Consequently, health literacy delivered through the peer network of SHGs is likely to change health practices among marginalised women.

In adjusting for other socio-economic confounders, this study also found that across wealth quintiles, a significant negative effect in BPCR practices was observed only in the poorest (fifth quintile) households, with these households least likely to practise BPCR.

Study strengths and limitations

This was a first-of-its-kind study in rural India that comprehensively evaluated the impact of an integrated microfinance and health literacy program on uptake of BPCR practice which can reduce delays in maternal care-seeking during complications.

A key strength of the study relates to the large sample size of 17244 women representing marginalised rural populations compared to other studies. Additionally, the study adds value to community evaluation research by using a quasi-experimental study design with pre-and post-intervention measurements using a valid comparator. The study design is appropriate for a high population low resource setting and provides the opportunity to evaluate the incremental change across program exposure levels (impact of SHG only, SHG plus health intervention), and the

For peer review only - http://bmjopen.bmj.com/site/about/guidelines.xhtml

potential diffusion effect (impact of SHG plus health intervention from members to non-members in areas without any program exposure).

Investigating the diffusion effect of practice from groups who received the intervention to neighbouring non-member households generated evidence on the expanded reach of such an intervention, leading to greater coverage of vulnerable populations. The key findings, however, should be interpreted while considering the following study limitations.

First, the selection of program blocks under the IMFHL was based on the program's operational criterion, thus preventing randomisation. The quasi-experimental survey design, however, adopted a multistage sampling approach using different criteria to select blocks (SC/ST percentage) and villages (SHG coverage and population size) to limit selection bias and allow for comparisons of areas with similar socio-cultural and economic characteristics.

Also, the survey collected information from women based on self-recall, which may suffer from recall bias and social desirability. However, the potential for these errors is minimised as the women were interviewed within 12 months post-delivery and by trained interviewers. Finally, while the survey design selected women from SHG member and non-member households from the same villages, in some cases, the women sampled in a given village would be either all members or all non-members, which potentially limited the variation in the outcome variable within villages.

Policy and program implications

A programmatic strength of the IMFHL intervention was that it utilised the existing structure of the SHG program to embed the health literacy component, which provided evidence of a scalable model for disseminating health information. The findings reported in this study are

For peer review only
For peer review only - http://bmjopen.bmj.com/site/about/guidelines.xhtml

relevant for the rural Indian context, where the Indian Government is establishing a network of women-only SHGs as a national poverty alleviation program. Therefore, allowing the integration of the health intervention into SHG structures, as the IMFHL program created the opportunity to improve maternal health outcomes for the state and the country.

Lastly, as healthcare-seeking behaviours are shaped by cultural practices in community settings, the diffusion effect of health messages from members to non-members can reinforce desired health behaviours. Diffusion also increases program coverage of harder to reach populations. Future research is required to explore the pathways of the diffusion effect of knowledge and practice from members to non-members in program villages.

CONCLUSIONS

The integrated microfinance and health literacy (IMFHL) program evaluated in this study promoted behaviour changes amongst some of the most marginalised households in UP, India's most populous state and one of its most developmentally disadvantaged areas. The findings show that SHGs exert both a dissemination effect of planned health behaviour within members as well as a diffusion effect of the natural transfer of BPCR practice from members to non-members but only when a health literacy component is added to SHGs. The findings from this research support integrating health literacy components into SHG networks and similar community groups to promote households' adoption of complication readiness plans to reduce delays in seeking health care during maternal complications.

Lessons from the IMFHL program implementation in India provide the opportunity to adapt SHG models implemented elsewhere by adding health literacy components. Beyond India, microfinance is promoted globally as a poverty alleviation program in low resource settings where the burden of maternal mortality remains high. These types of interventions' can assist

For peer review only - http://bmjopen.bmj.com/site/about/guidelines.xhtml

other low resource settings in accelerating progress towards the maternal health target of the Sustainable Development Goals.

Declarations

Acknowledgments

We wish to thank Professor Rachel Davey, Health Research Institute, the University of Canberra, for her guidance and for reviewing the final draft of this manuscript. The authors would also like to thank the University of Canberra (Australia), the Public Health Foundation of India (India) and the Population Council (India) for supporting this international research collaboration.

Author Contributions

DA conceptualised the study, conducted the analysis, interpreted the results and drafted the study. IM guided the study design, the data analysis, the interpretation of the results and discussion. IM provided substantive feedback and reviewed all drafts of the paper. TN guided the study design, the data analysis and interpretation of results and discussion. TN provided substantive feedback and reviewed all drafts of the paper. All authors read and approved the final manuscript.

Competing Interests

DA, IM and TN have no competing interests.

Funding

The paper is part of the PhD research of the first author (Danish Ahmad) who was supported by the University of Canberra's Higher Degree by Research scholarship for his PhD. Open

For peer review only - http://bmjopen.bmj.com/site/about/guidelines.xhtml

access funding for the publication is provided by the Bill and Melinda Gates Foundation who also funded the IMFHL program evaluated in the paper.

Role of Funders.

The funders had no role in study design, data collection and analysis, decision to publish, or preparation of the manuscript.

Availability of data and materials

The datasets used and/or analysed during the current study are available from the corresponding author on reasonable request.

Ethics Statement

Ethics approval was granted by the Human Research Ethics Committee at the University of Canberra. An independent ethics committee from the evaluation partner previously approved the primary data collection under the IMFHL project.

References

1. Berman P, Requejo J, Bhutta ZA, Singh NS, Owen H, Lawn JE. Countries' progress for women's and children's health in the Millennium Development Goal era: the Countdown to 2015 experience. BMC Public Health [Internet]. 2016;16(S2):791. Available from: http://bmcpublichealth.biomedcentral.com/articles/10.1186/s12889-016-3398-x

2. Moucheraud C, Owen H, Singh NS, Ng CK, Requejo J, Lawn JE, et al. Countdown to 2015 country case studies: what have we learned about processes and progress towards

For peer review only - http://bmjopen.bmj.com/site/about/guidelines.xhtml

MDGs 4 and 5? BMC Public Health [Internet]. 2016 [cited 2017 Oct 19];16(2). https://doi.org/10.1186/s12889-016-3401-6

3. United Nations. The Millennium Development Goals Report 2015 [internet]. New York: United Nations; 2015 [cited 2021 Jun 04]. Available from https://www.un.org/millenniumgoals/reports.shtml.

4. Alkema L, Chou D, Hogan D, Zhang S, Moller AB, Gemmill A, et al. Global, regional, and national levels and trends in maternal mortality between 1990 and 2015, with scenario-based projections to 2030: A systematic analysis by the un Maternal Mortality Estimation Inter-Agency Group. Lancet [Internet]. 2016;387(10017):462–74. Available from: http://dx.doi.org/10.1016/S0140-6736(15)00120-8

5. Department of Economic and Social Affairs, United Nations. The Sustainable Development Goals Report [Internet]. United Nations. New York; 2016. Available from: https://unstats.un.org/sdgs/report/2019/The-Sustainable-Development-Goals-Report-2019.pdf

6. Trends in maternal mortality 2000 to 2017: estimates by WHO, UNICEF, UNFPA, World Bank Group and the United Nations Population Division. [Internet]. World Health Organization. Geneva; 2019. Available from: https://www.who.int/reproductivehealth/publications/maternal-mortality-2000-2017/en/

7. Ronsmans, C., & Graham W. Maternal Survival 1 Maternal mortality : who, when , where, and why. Lancet [Internet]. 2006;368(9542):1189–200. Available from: https://www.thelancet.com/journals/lancet/article/PIIS014067360669380X/fulltext

For peer review only

For peer review only - http://bmjopen.bmj.com/site/about/guidelines.xhtml

8. Gowda SD. Millennium Development Goals - Final Country Report of India. 2017;(September):222. Available from: http://www.mospi.gov.in/sites/default/files/publication_reports/MDG_Final_Country_report_of_India_27nov17.pdf%0Awww.mospi.gov.in

9. Kassebaum NJ, Barber RM, Bhutta ZA, Dandona L, Gething PW, Hay SI, et al. Global, regional, and national levels of maternal mortality, 1990–2015: a systematic analysis for the Global Burden of Disease Study 2015. Lancet [Internet]. 2016;388(10053):1775–812. Available from: http://linkinghub.elsevier.com/retrieve/pii/S0140673616314702

10. Office of The Registrar General of India. Special Bulletin on Maternal Mortality in India 2015-17 [Internet]. 2019. Available from: http://www.censusindia.gov.in/vital_statistics/SRS_Bulletins/MMR_Bulletin-2015-17.pdf

11. Koblinsky M, Moyer CA, Calvert C, Campbell J, Campbell OMR, Feigl AB, et al. Quality maternity care for every woman, everywhere: a call to action. Lancet [Internet]. 2016;388(10057):2307–20. Available from: http://dx.doi.org/10.1016/S0140-6736(16)31333-2

12. Graham W, Woodd S, Byass P, Filippi V, Gon G, Virgo S, et al. Diversity and divergence: the dynamic burden of poor maternal health. Lancet [Internet]. 2016;388(10056):2164–75. Available from: http://dx.doi.org/10.1016/S0140-6736(16)31533-1

13. World Health Organization. WHO recommendations on health promotion interventions

For peer review only - http://bmjopen.bmj.com/site/about/guidelines.xhtml

for maternal and newborn health[internet]. Geneva: World Health Organization; 2015[cited 2021 Ap 16]. Available from: ttps://www.who.int/maternal_child_adolescent/documents/health-promotion-interventions/en

14. Solnes Miltenburg A, Roggeveen Y, Roosmalen J, Smith H. Factors influencing implementation of interventions to promote birth preparedness and complication readiness. BMC Pregnancy Childbirth. 2017;17(1):1–17.

15. Agarwal S, Sethi V, Srivastava K, Jha PK, Baqui AH. Birth preparedness and complication readiness among slum women in Indore city, India. Journal of Health Population and Nutrition. 2010;28(4):383–91.

16. Mbalinda SN, Nakimuli A, Kakaire O, Osinde MO, Kakande N, Kaye DK. Does knowledge of danger signs of pregnancy predict birth preparedness? A critique of the evidence from women admitted with pregnancy complications. Health Research and Policy Systems. 2014;12(1):1–7.

17. Campbell O, Graham WJ. Maternal Survival 2 Strategies for reducing maternal mortality : getting on with what works. 2006;368:1284–99. Available from: https://pubmed.ncbi.nlm.nih.gov/17027735/

18. Markos, Desalegn Bogale D. Birth preparedness and complication readiness among women of child bearing age group in Goba woreda, Oromia region, Ethiopia. BMC Pregnancy Childbirth [Internet]. 2014;14(1):1–9. Available from: http://www.embase.com/search/results?subaction=viewrecord&from=export&id=L600

For peer review only - http://bmjopen.bmj.com/site/about/guidelines.xhtml

014040%5Cnhttp://dx.doi.org/10.1186/1471-2393-14-282

19. Mbachu II, Ezeama C, Osuagwu K, Umeononihu OS, Obiannika C, Ezeama N. A cross sectional study of maternal near miss and mortality at a rural tertiary centre in southern nigeria. BMC Pregnancy Childbirth. 2017;17(1):1–8.

20. Kananura RM, Tetui M, Bua J, Ekirapa-Kiracho E, Mutebi A, Namazzi G, et al. effect of a participatory multisectoral maternal and newborn intervention on birth preparedness and knowledge of maternal and newborn danger signs among women in Eastern Uganda: A quasi-experiment study. Glob Health Action [Internet]. 2017;10(0). Available from: https://doi.org/10.1080/16549716.2017.1362826

21. Countdown to 2015. Maternal, New Born and Child Survival: India 2015 report. 2015;(000):142–3.

22. Mission of Health and Family Welfare. 10th Common review mission [Internet]. 2016. Available from: https://nhm.gov.in/index1.php?lang=1&level=2&sublinkid=825&lid=448

23. Saxena D, Vangani R, Mavalankar D V., Thomsen S. Inequity in maternal health care service utilisation in Gujarat: Analyses of district-level health survey data. Glob Health Action. 2013;6(1). doi:10.3402/gha.v6i0.19652

24. Knight HE, Self A, Kennedy SH. Why Are Women Dying When They Reach Hospital on Time? A Systematic Review of the "Third Delay." PLoS One. 2013;8(5). doi: 10.1371/journal.pone.0063846

25. Sri S, Khanna R, Abhiyan JS. Dead Women Talking [Internet]. Dead Women Talking.

For peer review only - http://bmjopen.bmj.com/site/about/guidelines.xhtml

2018 [cited 2021 May 9]. Available from: http://www.commonhealth.in/Dead Women Talking full report final.pdf

26. Gabrysch S, Campbell OMR. Still too far to walk: Literature review of the determinants of delivery service use. BMC Pregnancy Childbirth [Internet]. 2009;9(1):34. Available from: https://doi.org/10.1186/1471-2393-9-34

27. Chaturvedi S, Randive B, Diwan V, De Costa A. Quality of obstetric referral services in India's JSY cash transfer programme for institutional births: A study from Madhya Pradesh province. PLoS One. 2014;9(5). https://doi.org/10.1371/journal.pone.0096773

28. Institute for Health Metrics and Evaluation (IHME). GBD PROFILE : INDIA. Global Burden of Disease Study 2010 (GBD 2010). 2010;(GBD):1–4. Available from: http://www.healthdata.org/sites/default/files/files/country_profiles/GBD/ihme_gbd_country_report_india.pdf.

29. United Nations. India and the MDGs Towards a sustainable future for all. 2015.

30. Sri S, Khanna R, Abhiyan JS. Dead Women Talking A civil society report on maternal deaths in India People's Health Movement -India. [cited 2017 Nov 12]; Available from: http://www.commonhealth.in/Dead Women Talking full report final.pdf

31. Kawachi I, Kennedy BP, Glass R. Social capital and self-rated health: A contextual analysis. Am J Public Health. 1999;89(8):1187–93

32. Manandhar et al. Articles Effect of a participatory intervention with women's groups on birth outcomes in Nepal: cluster-randomised controlled trial. www.thelancet.com

For peer review only - http://bmjopen.bmj.com/site/about/guidelines.xhtml

[Internet]. 2004 [cited 2017 Dec 2];364(11). https://doi.org/10.1016/S0140-6736(04)17021-9

33. Kumar V, Kumar A, Ghosh AK, Samphel R, Yadav R, Yeung D, et al. Enculturating science: Community-centric design of behavior change interactions for accelerating health impact. Semin Perinatol [Internet]. 2015;39(5):393–415. Available from: http://dx.doi.org/10.1053/j.semperi.2015.06.010

34. Baqui AH, Williams EK, Rosecrans AM, Agrawal PK, Ahmed S, Darmstadt GL, et al. Impact of an integrated nutrition and health programme on neonatal mortality in rural northern India. Bull World Health Organ. 2008;86(10):796–804.

35. Kumar V, Mohanty S, Kumar A, Misra RP, Santosham M, Awasthi S, et al. Effect of community-based behaviour change management on neonatal mortality in Shivgarh, Uttar Pradesh, India: a cluster-randomised controlled trial. Lancet. 2008;372(9644):1151–62.

36. Prost A, Colbourn T, Seward N, Azad K, Coomarasamy A, Copas A, et al. Women's groups practising participatory learning and action to improve maternal and newborn health in low-resource settings: A systematic review and meta-analysis. Lancet [Internet]. 2013;381(9879):1736–46. Available from: http://dx.doi.org/10.1016/S0140-6736(13)60685-6

37. Gage AJ. Barriers to the utilisation of maternal health care in rural Mali. Soc Sci Med. 2007;65(8):1666–82. https://doi.org/10.1016/j.socscimed.2007.06.001

38. Ahmad D, Mohanty I, Hazra A, Niyonsenga T. The Knowledge of Danger Signs of

For peer review only - http://bmjopen.bmj.com/site/about/guidelines.xhtml

Obstetric Complications Among Women in Rural India : Evaluating an Integrated Microfinance and Health Literacy Program. BMC Pregnancy Childbirth 21, 79 (2021). https://doi.org/10.1186/s12884-021-03563-

39. Hazra A, Atmavilas Y, Hay K, Saggurti N, Verma RK, Ahmad J, et al. Effects of health behaviour change intervention through women's self-help groups on maternal and newborn health practices and related inequalities in rural india: A quasi-experimental study. EClinicalMedicine [Internet]. 2020;18:100198. Available from: https://doi.org/10.1016/j.eclinm.2019.10.011

40. Singh R, Neogi SB, Hazra A, Irani L, Ruducha J, Ahmad D, Kumar S, Mann N, Mavalankar D. Utilization of maternal health services and its determinants: a cross-sectional study among women in rural Uttar Pradesh, India. J Heal Popul Nutr [Internet]. 2019;38(13):1–12. Available from: http://doi.org/10.1186/s41043-019-0173-5

41. Lyngdoh T, Neogi SB, Ahmad D, Soundararajan S, Mavalankar D. Intensity of contact with frontline workers and its influence on maternal and newborn health behaviors: cross-sectional survey in rural Uttar Pradesh, India. J Health Popul Nutr. 2018;37(1):2. Published 2018 Jan 8. doi:10.1186/s41043-017-0129-6

42. Ruducha J, Hariharan D, Potter J, et al.Measuring coordination between women's self-help groups and local health systems in rural India: a social network analysis. BMJ Open 2019;9:e028943. doi: 10.1136/bmjopen-2019-028943

43. Census of India : Provisional Population Totals Paper 1 of 2011 : Uttar Pradesh

For peer review only - http://bmjopen.bmj.com/site/about/guidelines.xhtml

[Internet]. [cited 2021 May 30]. Available from: http://www.censusindia.gov.in/2011-prov-results/prov_data_products_up.html

44. Ruducha J, Huang X, Potter J, Hariharan D, Ahmad D, Kumar S, et al. Perceived Social Networks and Newborn Health: Evidence from Self-Help Group Communities in Northern India. Societies [Internet]. 2018;8(4):92. Available from: http://doi.org/10.3390/soc8040092

45. Devasenapathy N, Neogi SB, Soundararajan S, Ahmad D, Hazra A, Ahmad J, et al. Association of antenatal care and place of delivery with newborn care practices: evidence from a cross-sectional survey in rural Uttar Pradesh, India. J Heal Popul Nutr [Internet]. 2017;36(1):30. Available from: http://doi.org/10.1186/s41043-017-0107-z

46. Walia M, Irani L, Chaudhuri I, Atmavilas Y, Saggurti N. Effect of sharing health messages on antenatal care behavior among women involved in microfinance-based self-help groups in Bihar India. Glob Heal Res Policy. 2020;5(1):1–8.

For peer review only

For peer review only - http://bmjopen.bmj.com/site/about/guidelines.xhtml

STROBE Statement—Checklist of items that should be included in reports of ***case-control studies***

	Item No	Recommendation	Page No
**Title and abstract**	1	(*a*) Indicate the study's design with a commonly used term in the title or the abstract	2
		(*b*) Provide in the abstract an informative and balanced summary of what was done and what was found	2
**Introduction**			
Background/rationale	2	Explain the scientific background and rationale for the investigation being reported	3
Objectives	3	State specific objectives, including any prespecified hypotheses	6-7
**Methods**			
Study design	4	Present key elements of study design early in the paper	8-9
Setting	5	Describe the setting, locations, and relevant dates, including periods of recruitment, exposure, follow-up, and data collection	8-9
Participants	6	(*a*) Give the eligibility criteria, and the sources and methods of case ascertainment and control selection. Give the rationale for the choice of cases and controls	7
		(*b*) For matched studies, give matching criteria and the number of controls per case	8(references 38,39)
Variables	7	Clearly define all outcomes, exposures, predictors, potential confounders, and effect modifiers. Give diagnostic criteria, if applicable	10
Data sources/ measurement	8*	For each variable of interest, give sources of data and details of methods of assessment (measurement). Describe comparability of assessment methods if there is more than one group	10-11
Bias	9	Describe any efforts to address potential sources of bias	25-26
Study size	10	Explain how the study size was arrived at	
Quantitative variables	11	Explain how quantitative variables were handled in the analyses. If applicable, describe which groupings were chosen and why	10-11
Statistical methods	12	(*a*) Describe all statistical methods, including those used to control for confounding	10-11
		(*b*) Describe any methods used to examine subgroups and interactions	10-11
		(*c*) Explain how missing data were addressed	na
		(*d*) If applicable, explain how matching of cases and controls was addressed	10-11
		(*e*) Describe any sensitivity analyses	na
**Results**			
Participants	13*	(a) Report numbers of individuals at each stage of study—eg numbers potentially eligible, examined for eligibility, confirmed eligible, included in the study, completing follow-up, and analysed	16-19
		(b) Give reasons for non-participation at each stage	16-19
		(c) Consider use of a flow diagram	8
Descriptive data	14*	(a) Give characteristics of study participants (eg demographic, clinical, social) and information on exposures and potential confounders	12
		(b) Indicate number of participants with missing data for each variable of interest	12

For peer review only

Outcome data	15*	Report numbers in each exposure category, or summary measures of exposure	12

For peer review only

Main results	16	(*a*) Give unadjusted estimates and, if applicable, confounder-adjusted estimates and their precision (eg, 95% confidence interval). Make clear which confounders were adjusted for and why they were included	
		(*b*) Report category boundaries when continuous variables were categorized	
		(*c*) If relevant, consider translating estimates of relative risk into absolute risk for a meaningful time period	
Other analyses	17	Report other analyses done—eg analyses of subgroups and interactions, and sensitivity analyses	
**Discussion**			
Key results	18	Summarise key results with reference to study objectives	
Limitations	19	Discuss limitations of the study, taking into account sources of potential bias or imprecision. Discuss both direction and magnitude of any potential bias	
Interpretation	20	Give a cautious overall interpretation of results considering objectives, limitations, multiplicity of analyses, results from similar studies, and other relevant evidence	
Generalisability	21	Discuss the generalisability (external validity) of the study results	
**Other information**			
Funding	22	Give the source of funding and the role of the funders for the present study and, if applicable, for the original study on which the present article is based	

*Give information separately for cases and controls.

Note: An Explanation and Elaboration article discusses each checklist item and gives methodological background and published examples of transparent reporting. The STROBE checklist is best used in conjunction with this article (freely available on the Web sites of PLoS Medicine at http://www.plosmedicine.org/, Annals of Internal Medicine at http://www.annals.org/, and Epidemiology at http://www.epidem.com/). Information on the STROBE Initiative is available at http://www.strobe-statement.org.

For peer review only

For peer review only - http://bmjopen.bmj.com/site/about/guidelines.xhtml

BMJ Open

Improving birth preparedness and complication readiness in rural India through an integrated microfinance and health literacy program: Evidence from a quasi-experimental study

Journal:	*BMJ Open*
Manuscript ID	bmjopen-2021-054318.R1
Article Type:	Original research
Date Submitted by the Author:	30-Nov-2021
Complete List of Authors:	Ahmad, Danish; University of Canberra, Health Research Institute,Faculty of Health; Indian Institute of Public Health Gandhinagar (IIPH-G)<br>Mohanty, Itismita ; University of Canberra, Health Research Institute, Faculty of Health<br>Niyonsenga, Theophile; University of Canberra, Health Research Institute, Faculty of Health
**Primary Subject Heading**:	Public health
Secondary Subject Heading:	Health services research
Keywords:	Maternal medicine < OBSTETRICS, PUBLIC HEALTH, EPIDEMIOLOGY, OBSTETRICS, International health services < HEALTH SERVICES ADMINISTRATION & MANAGEMENT

I, the Submitting Author has the right to grant and does grant on behalf of all authors of the Work (as defined in the below author licence), an exclusive licence and/or a non-exclusive licence for contributions from authors who are: i) UK Crown employees; ii) where BMJ has agreed a CC-BY licence shall apply, and/or iii) in accordance with the terms applicable for US Federal Government officers or employees acting as part of their official duties; on a worldwide, perpetual, irrevocable, royalty-free basis to BMJ Publishing Group Ltd ("BMJ") its licensees and where the relevant Journal is co-owned by BMJ to the co-owners of the Journal, to publish the Work in this journal and any other BMJ products and to exploit all rights, as set out in our licence.

The Submitting Author accepts and understands that any supply made under these terms is made by BMJ to the Submitting Author unless you are acting as an employee on behalf of your employer or a postgraduate student of an affiliated institution which is paying any applicable article publishing charge ("APC") for Open Access articles. Where the Submitting Author wishes to make the Work available on an Open Access basis (and intends to pay the relevant APC), the terms of reuse of such Open Access shall be governed by a Creative Commons licence – details of these licences and which Creative Commons licence will apply to this Work are set out in our licence referred to above.

Other than as permitted in any relevant BMJ Author's Self Archiving Policies, I confirm this Work has not been accepted for publication elsewhere, is not being considered for publication elsewhere and does not duplicate material already published. I confirm all authors consent to publication of this Work and authorise the granting of this licence.

For peer review only

Improving birth preparedness and complication readiness in rural India through an integrated microfinance and health literacy program: Evidence from a quasi-experimental study

Danish Ahmad (PhD)[1,2*], Itismita Mohanty (PhD)[1], Theophile Niyonsenga(PhD)[1]

1. Health Research Institute, Faculty of Health, University of Canberra, Canberra, Australia
2. Public Health Foundation of India, and Indian Institute of Public Health Gandhinagar (IIPH-G), Gujarat, India

*Corresponding author
Health Research Institute,
Faculty of Health, University of Canberra,
Canberra, Australia
E-mail: drdanish.research@gmail.com
Word count: 4975

For peer review only

Abstract:

Objective: Recently, a novel community health program — the integrated microfinance and health literacy (IMFHL) program was implemented through microfinance-based women's only self-help groups (SHGs) in India to promote birth preparedness and complication readiness (BPCR) to improve maternal health. The study evaluated the impact of the IMFHL program on BPCR practice by women in one of India's poorest states — Uttar Pradesh — adjusting for the community, household and individual variables. The paper also examined for any diffusion of knowledge of BPCR from SHG members receiving the health literacy intervention to non-members in program villages. **Design**: Quasi-experimental study using cross-sectional survey data. **Settings:** Secondary survey data from the IMFHL program was used. **Participants:** Survey data was collected from 17,244 women in households with SHG member and non-member households in rural India. **Primary outcomes:** Multivariable logistic regression was used to estimate main and adjusted IMFHL program effects on maternal BPCR practice in their last pregnancy. **Results:** Membership in SHGs alone is positively associated with BPCR practice, with 17 per cent higher odds(OR=1.17, 95% CI: 1.07–1.29, p<0.01) of these women practising BPCR compared to women in villages without the programs. Furthermore, the odds of practising complete BPCR increase to almost fifty per cent(OR=1.48, 95% CI: 1.35–1.63, p<0.01) when a maternal health literacy component is added to the SHGs. A diffusion effect was found for BPCR practice from SHG members to non-members when the health literacy component was integrated into the SHG model. **Conclusions:** The results suggest that SHG membership exerts a positive impact on planned health behaviour and a diffusion effect of BPCR practice from members to non-members when SHGs are enriched with a health literacy component. The study provides evidence to guide the implementation of community health programs seeking to promote BPCR practise in low resource settings.

For peer review only - http://bmjopen.bmj.com/site/about/guidelines.xhtml

Study Strengths and Limitations.

- This is the first study to evaluate the potential of delivering maternal health literacy for pregnancy complications at scale through women based microfinance groups to rural low-income communities where higher maternal health inequities persist.
- The study's evaluation framework and research question adapted the widely used three delays model and the established social determinants of health framework.
- The evaluation design used a novel methodology to identify the program's main effect and adjusted effects using a comprehensive list of variables using a multivariate stepwise forward selection process to examine how program-associated outcomes are influenced.
- The study was limited by a lack of randomisation in the selection of the survey blocks during program implementation owing to pragmatic operational considerations in a high-population and low-resource setting.

Introduction

Improving maternal health is a global priority in the attainment of the Sustainable Development Goals (SDGs). Overall, health system reforms and socio-economic improvements in low resource countries led to an almost fifty per cent reduction in maternal deaths by the end of the Millennium Development Goals (MDGs) in 2015 compared to the 1990s (1–3). However, the rate of decline was inadequate to meet the global MDG target of a 75 per cent reduction in the maternal mortality rate (MMR) (3,4). Reflecting this, more than 295,000 maternal deaths occurred in 2017, mostly due to preventable causes across the maternity period, including hypertensive disorders of pregnancy, severe bleeding, obstructed labour and sepsis (4–6), all conditions with established treatment options. The high number of avoidable deaths stemming from these causes reflects the residual gap between the MDG target and available means to

For peer review only

For peer review only - http://bmjopen.bmj.com/site/about/guidelines.xhtml

achieve it. The SDG has a further stringent target of reducing the MMR to 70 per 100,000 live births by 2030 for all countries (5). However, the current situation has been further challenged by severe health service disruptions due to COVID-19. Achieving the SDGs' maternal health target requires concerted action in select high poverty and low resource countries in Sub-Saharan Africa and South Asia, where almost 99 per cent of global preventable maternal deaths occur (1,7,8). India has the world's second-highest number of maternal deaths, with almost 45,000 maternal deaths annually (9,10). Although India invested substantially in improving health service delivery for its predominantly rural populations, high maternal deaths remain a challenge (6,8). Attaining the SDG target requires specific improvements in India to accelerate maternal mortality decline beyond that observed in the MDG era and the adoption of novel strategies that bridge health system inadequacies with civil society actions (11,12).

While India has prioritised institutional delivery through a national conditional cash transfer scheme incentivising deliveries in a health facility, substantial gaps persist in early identification and care of maternal complications, especially in the antenatal and postnatal period for rural populations (10,13,14). Inadequate referral services and limited emergency obstetric care (EmOC) place women at higher risk of adverse maternal outcomes in rural areas (12, 13,14). Health system inadequacies are further compounded by community-related delays in seeking care and reaching health facilities due to financial barriers and lack of birth and complication readiness (13,15-19). Maternal death reviews have shown that high poverty and low development states in the north of India, especially Uttar Pradesh, account for the majority of maternal deaths (8,10,20).

Studies from India and rural Uttar Pradesh (UP) have shown that, despite higher institutional deliveries, deficiencies in antenatal care (ANC) limit the transmission of key maternal health

For peer review only - http://bmjopen.bmj.com/site/about/guidelines.xhtml

information such as birth preparedness and complication readiness (BPCR) to pregnant women (14,21). Improving birth preparedness and complication readiness (BPCR) is strategic to reduce maternal mortality through increasing awareness and preparedness around emergency obstetrics care (13). Pregnant women and their households are expected to receive BPCR advice as part of antenatal care services provided by health workers (13). In a rural setting, irrespective of risk, where antenatal and referral services are inadequate to ensure monitoring of pregnancies, households' adoption of BPCR assumes greater importance (14,21,22).

Additionally, studies have shown that BPCR is challenging to achieve in low resource settings where health system services are lacking, and households have low education and high poverty levels (2,12,23–27). Recently, a promising community-based intervention that builds on the concept of empowering poor rural pregnant women by providing them with health literacy and finance-based assistance through women-only microfinance groups was implemented in rural UP. The integrated microfinance and health literacy (IMFHL) program was built on other community-based programs that have shown an improvement in routine indicators of maternal health (28–31)implemented on the microfinance platform to provide pregnant and recently delivered women with health messages. Information is provided to improve knowledge of dangerous signs related to pregnancy, childbirth and post-delivery complications and to adopt birth preparedness and complication readiness practice in order to reduce delays associated with seeking care. Microfinance-based SHG platforms in UP and other low-development regions of India have previously shown that integrated health literacy and SHG interventions can improve routine maternal health practices such as antenatal care utilisation, increase institutional deliveries and also improve newborn health practises at home, such as cord care and timely breastfeeding(32,33). However, the impact of membership in a SHG-based health program on the practice of BPCR among SHG members to reduce maternal mortality has not been

For peer review only - http://bmjopen.bmj.com/site/about/guidelines.xhtml

evaluated. Also, the potential of the program to diffuse health information from members to non-members in villages where SHGs are established is untested.

This research builds on limited evidence on the effectiveness of membership in microfinance programs integrated with a health literacy component on birth preparedness and complication readiness practice among women in rural areas (32–36). It is hypothesised that women in low-income rural households receiving financial access and additional health information through self-help groups (SHGs) would reduce healthcare-seeking delays during maternal complications.

This study evaluated the impact of membership in an integrated microfinance and health literacy (IMFHL) program on the practice of birth preparedness and complication readiness (BPCR) by women during their last pregnancy in rural UP. It also evaluated whether the IMFHL program contributed to the adoption of BPCR among women who did not participate in the IMFHL program as SHG members through a process of diffusion of behaviours from SHG members to neighbouring non-member households in the same villages where the program was implemented.

METHODS

The study used cross-sectional data from a quasi-experimental survey design, collected as part of the IMFHL program in two rounds (pre-post program implementation), capturing the program's characteristics and other factors in the first round in 2015, followed by a second round of data collection two years into the program in 2017 (32,33).

Description of the IMFHL program intervention

The IMFHL program was implemented over five years (2012–2017) in two phases: an initial trial or learning phase in limited areas from 2012- 2015, followed by at-scale implementation

For peer review only - http://bmjopen.bmj.com/site/about/guidelines.xhtml

from 2015-2017(32,33). The program developed the final implementation design and scale-up strategy based on experiences in the pilot phase. At scale, program implementation occurred from 2015-2017 in UP state, and the survey data collected at the start and end of this period is used for this paper(32,33).

The maternal and neonatal health literacy component was layered upon a pre-existing microfinance-based SHG platform towards 2015 to provide pregnant and recently delivered women with health messages in rural UP. A detailed description of the IMFHL program context, selection of intervention, comparison and control blocks is available elsewhere (32,33).

A woman was classified as a SHG member if she was a member herself or someone else in the household, usually a mother-in-law or sister-in-law, was a member (32). These SHG members represented the first tier of beneficiaries who directly received health information, encouraging them to adopt desired health behaviours to promote maternal and neonatal health. As members and non-members lived in the same villages, it was expected that members receiving health information would communicate knowledge to neighbouring non-member households through informal communication networks that would gradually lead to the adoption of desired behaviours, as observed elsewhere in previous social network analysis studies (36,37). The process of this social transfer of health information and practices through informal networking to non-members is known as the diffusion effect, also evaluated in this paper (32,36,37). An underlying process of collective socialisation (32,37) is expected to explain the diffusion process in which health literate SHG members serve as role models to help non-members in their community adopt protective behaviours around pregnancy and childbirth for safer maternal outcomes. This diffusion of knowledge from members (tier I) to neighbouring non-members (tier II) is depicted in Figure 1 below.

For peer review only - http://bmjopen.bmj.com/site/about/guidelines.xhtml

FIGURE 1

Study setting and participants

Sampling procedure

The survey sampling design followed UP's administrative hierarchy and collected data from households in *gram panchayats* (GPs) or villages from 70 blocks in 20 districts, aiming for a representative sample from the program's coverage area (32,38). Moreover, while the same GPs, blocks and districts were visited in both survey rounds, different households were sampled and interviewed in each round. The sampling used a multistage stratified sampling design to select blocks, GPs and households across three areas based on the IMFHL program's exposure: intervention(SHG plus health implemented), comparator(SHG only implemented) and pure control (neither SHG nor health implemented) areas. A detailed description of the selection strategy is available elsewhere (32,33).

Sample size and data

The analytical sample used in this study comprised a total of 17,244 eligible women, of whom 59 per cent (10,097 women) did not belong to SHGs. The data was collected at the individual, household and community levels using separate structured questionnaires. The survey collected data from currently married women aged 15 to 49 years who had delivered an infant in the 12 months preceding the survey and from the household head and village representatives (32, 33). Thus eligible women interviewed in round 1(2015) would have delivered in the 12 months before the program's start, while those women interviewed in round 2(2017) would have delivered after the health intervention was started. The total sampling size was estimated with

For peer review only - http://bmjopen.bmj.com/site/about/guidelines.xhtml

a 85% power, the usual 5% level of significance and a design effect of 2. The detailed sample size determination is reported elsewhere (32,33).

Outcome, exposure and confounding variables

Outcome variable:

The birth preparedness and complication readiness (BPCR) practice captured eligible women's self-reported BPCR steps taken during the last pregnancy that would have occurred 12 months prior to the survey round(s). The outcome variable, a binary variable, was constructed with '0' representing partial or no BPCR preparation and '1' representing complete BPCR, defined as households that practised all eight birth preparedness and complication readiness steps during the last pregnancy as outlined by the World Health Organization. In both survey rounds, eligible women, independent of the place of delivery, were asked to recall multiple responses to the question: "*What advance preparation did you/your family members make to manage in case of any pregnancy/delivery complications?"*. The interview responses were marked against eight key steps required to achieve BPCR such as: (1) 'Decided on the place of delivery—home or health facility'; (2) 'Knew the facility that could provide emergency care'; (3) 'Identified institution where to rush in case of emergency'; (4) 'Identified people to accompany the woman', (5) 'Identified people to take care of children at home'; (6) 'Saved/arranged money for delivery expense or in case of emergency'; (7) 'Advance arrangement of transportation to go to the facility' and (8) 'others—cloth, soap'.

Main exposure variable.

The exposure variable, the IMFHL intervention, comprised four levels based on households' exposure to the IMFHL program. An ordinal variable was created allowing us to evaluate the program's main effect on the practice of BPCR, that is, the magnitude of change in BPCR practice across levels of IMFHL program exposure: intervention (SHG plus health),

For peer review only - http://bmjopen.bmj.com/site/about/guidelines.xhtml

comparison (SHG only) and pure control (no SHG, no health) households. The coding of the IMFHL explanatory variable, with a description of each group, is shown below:

- **Group 0:** households that were not SHG members (non-members) and were in villages without *any* program intervention (pure control households).
- **Group 1**: households that were not SHG members (non-members) but were in *program villages* where either the SHG program alone or SHG program plus the health intervention was implemented (diffusion-control households).
- **Group 2**: households that were SHG members in program villages where only the SHG program was implemented (comparison households).
- **Group 3**: households that were SHG members and were in villages where both the SHG program and the additional health intervention was provided. Only these households received health literacy intervention through the SHG (intervention households).

Additionally, a survey round variable was created to assess the effect of the intervention program over time (change in women's BPCR in round II in 2017 compared to round I in 2015, when the program was yet to be implemented).

Confounding variables:

A comprehensive set of confounding variables capturing individual, household and community-level characteristics were identified from previous maternal and child health literature and included in models III and IV. The confounding variables in model III represented eligible women's maternal health status and broader maternal health service utilisation indicators. Additional socio-demographic and area-level characteristics were included in the final Model IV. Model IV also included a household wealth quintile variable, constructed for this analysis using Polychoric Principal Component Analysis (PCA), combining all household assets and amenities to evaluate BPCR association across five wealth gradients extending from marginally poor (reference category) to the poorest households. Covariates also included a variable to

For peer review only - http://bmjopen.bmj.com/site/about/guidelines.xhtml

capture household poverty using the income threshold of 'Below Poverty Line card(BPL)' set by the Indian Government.

Statistical analysis

A multivariable logistic regression modelling approach was used to evaluate the intervention program's impact on the level of birth preparedness and complication readiness practice among women in rural UP, adjusting for various confounding variables. The main explanatory variable, IMFHL intervention, was categorised according to four levels of household exposure to the IMFHL program, as briefly outlined below. Four separate regression models were fitted to the data, with the first model (Model I) establishing the program's main effect without any confounding variables but including the survey round variable. The second model (Model II) included an interaction term (IMFHL intervention by survey round) to draw out the change over time in the effect of IMFHL program exposure. Confounders related to individual health and health system were included in Model III, while the full model Model IV included socio-demographic, economic and area-level variables. Thus Models I and II provide unadjusted program effects while models III and IV provide adjusted effects. All analyses were performed using Stata 16 (Statacorp, USA).

Results

Descriptive Statistics

Table 1 compiles the levels and steps of birth preparedness and complication readiness (BPCR) practice in the last pregnancy among women aged 15–49 in rural UP (n=17,244). As Table 1 reveals, only 24 per cent of all women in this analysis had not practised any steps for birth or complication readiness, while 49 per cent have undertaken full BPCR in their last pregnancy. A further 6 per cent of women had only taken steps to address birth preparedness, while 21 per cent of all women had only undertaken the three steps required for complication readiness.

For peer review only - http://bmjopen.bmj.com/site/about/guidelines.xhtml

Table 1: Levels of BPCR practice in last pregnancy among women aged 15–49 in rural Uttar Pradesh

Level of preparedness	Number (%) (n=17,244)	Specific activity done
No BPCR preparedness	4,187 (24 %)	
Only birth preparedness done	955 (6%)	0. Decided on the place of delivery (at home or health facility)
		1. Knew the facility that could provide emergency care
		2. Identified people to accompany the woman
		3. Identified people to take care of children at home
		4. Others (oil/cloth/soap)
Only complication readiness done	3,697 (21%)	1. Saved/arranged money for delivery expense or in case of emergency
		2. Advance arrangement of transportation to go to the facility
		3. Identified institution where to rush in case of emergency
Both birth preparation and complication readiness steps done	8,405 (49%)	1. Decided on the place of delivery (at home or health facility)
		2. Knew the facility that could provide emergency care
		3. Identified people to accompany the woman
		4. Identified people to take care of children at home
		5. Saved/arranged money for delivery expense or in case of emergency
		6. Advance arrangement of transportation to go to the facility
		7. Identified institution where to rush in case of emergency
		8. Others (oil/cloth/soap)
**Total**	**17,244 (100%)**	

Notes - BPCR: birth preparedness and complication readiness.

Table 2 presents the key descriptive statistics for eligible women categorised across SHG member and non-member households across all variables used in this paper. The study sample included more women from non-member households (59 per cent) than from member households, but with an equal allocation of households within various levels of IMFHL program exposure.

Sampled women had a mean parity of 2.4 (median 2, range 0–9) which reflects the fertility rate in rural UP, but is higher than the current Indian fertility rate (median = 2.2, range: 1–4) (38).

For peer review only

Moreover, close to 48 per cent of all surveyed women reported experiencing a pregnancy complication in their last pregnancy, while only a quarter (25%) had suffered a spontaneous or induced abortion. Furthermore, women on average had four contacts with frontline workers in their last pregnancy, with 26 per cent of women receiving four ANC visits with all tests during their last pregnancy and only nine per cent of women receiving the vital three postnatal visits. The table compared SHG members and Non-members using Chisqure and Independent samples-T test with statistical significance reported. While comparability is seen for key variables, all descriptive variables were adjusted in subsequent regression models(III-IV).

Table 2: Summary statistics of key variables by non-member households and SHG households

		**Summary statistics (N=17,244)**		
	**Variable**	**Non- member households**	**SHG house holds**	**Test of Comparison**
	Independent variables: Program exposure characteristics			
1.	**Level of household (HH) microfinance (MF) exposure**	10,097(59%)	7,147(41%)	---
	0. HH in a village with **no SHG, no health** intervention (pure control – reference)	3709(37%)	---	---
	1. **Non-member** HH in a village with **SHG only program**	3042(30%)	---	---
	2. **Non-member** HH in a village with **SHG plus health** intervention	3346(33%)	---	---
	3. **SHG member** HH in a village with **SHG only intervention**	---	3623(51%)	---
	4. **SHG plus health member** HH in a village with **SHG *plus* health** intervention	---	3524(49%)	---
2.	**Evaluation survey round**			
	Round 1/ baseline-2015 (=0)	5,454 (54%)	3,269 (45%)	***
	Round 2/ endline-2017 (=1)	4,643 (45%)	3,878 (54%)	
	Independent variables: Individual health and health system characteristics			
3.	Parity (number of previous pregnancies) of the eligible woman (EW)	Mean=2.4 (SD=1.44)	Mean=2.4 (SD=1.41)	
4.	Ew with any past history of pregnancy loss (due to spontaneous/induced abortion)	2,550(25%)	1,890(26%)	*
5.	EW experienced any complication in last pregnancy/labour or post-partum	4,784 (47%)	3,437 (48%)	
6.	EW's with correct knowledge of the minimum(four) number of antenatal check-ups (ANC) required during pregnancy	3,716 (37%)	2,761 (39%)	***
7.	EW received four or more ANC in last pregnancy with urine/blood pressure /weight/abdominal/ultrasound tested in last ANC	2,668 (26%)	2,077 (29%)	***
8.	EW reporting Institutional delivery	8,357 (83%)	5948(83%)	

For peer review only - http://bmjopen.bmj.com/site/about/guidelines.xhtml

		Summary statistics (N=17,244)		
	**Variable**	**Non- member households**	**SHG house holds**	**Test of Comparison**
9.	**Duration (in hours) of stay in health facility immediately after delivery**			
	1. Home delivery (reference)	1,740 (17%)	1,199 (17%)	
	2. Discharged within 12 hrs	5,603 (56%)	4,127 (58%)	
	3. Discharged between 12–24 hrs	1,134 (11%)	795 (11%)	***
	4. Discharged between 24–48 hrs	720 (7%)	506 (7%)	
	5. Discharged between 48–72 hrs	283 (3%)	146 (2%)	
	6. Discharged after >72 hrs	617 (6%)	374 (5%)	
10.	EW who received three postnatal care visits (PNC) in the first seven days after delivery	909 (9%)	659 (9%)	
11.	Number of contacts with ASHA/ANM/AWW/SHG in last pregnancy	Mean=4.0 (SD=5.4)	Mean=4.2 (SD=5.4)	
12.	Distance (kilometres) to primary health centre if not available in the village	Mean=5.4 (SD=4.9)	Mean=5.4 (SD=4.7)	
	Independent variables: Socio-demographic/economic and area level characteristics			
13.	Village distance (kilometres) to closest town	Mean=1.4 (SD=0.77)	Mean=1.4 (SD=0.75)	
14.	Population of village	Mean=5,153 (SD=5,134)	Mean=5,140 (SD=5,113)	
15.	Household (HH) with Below Poverty Line (BPL) Card	4,499 (45%)	3316(46%)	
16.	**Household wealth quintile (poor to poorest)**			
	1. Marginally Poor	1,985 (20%)	1,498 (21%)	
	2. Moderately Poor	2,049 (20%)	1,502 (21%)	
	3. Poor	1,994 (20%)	1,471 (20%)	***
	4. Poorer	2,029 (20%)	1,396 (20%)	
	5. Poorest	2,040 (20%)	1,280 (18%)	
17.	EW presently working to earn cash, in kind or both	1,661 (16%)	1,211 (17%)	
18.	Ew living with joint and extended family	5,819 (58%)	4,259 (60%)	***
19.	Household head's regligion: Hinduism and others	9,281(92%)	6,546 (92%)	
20.	EW belonging to scheduled caste and scheduled tribe	4,485(45%)	3,202(45%)	
21.	EW age in completed years	Mean=25 (SD=4.56)	Mean=25 (SD=4.55)	
22.	EW's education level: completed primary/middle school (up to Year 9) and above	6,680(66%)	4,798(67%)	
23.	EW's husband education: completed primary/middle school (up to Year 9) and above	8,365 (83%)	5,936(83%)	
	Dependent variable			
24.	EW's who practised birth preparedness and complication readiness (BPCR) in last pregnancy	4,662(46%)	3,743(52%)	***

Acronyms: Eligible woman (EW), Self-help group (SHG), Accredited Social Health Worker (ASHA)/Auxiliary Nurse Midwife (ANM) Anganwadi worker (AWW). ASHA/ANM & AWW are government health workers in villages as per population guidelines providing preventative maternal, child and other health services. Independent sample T-test and Chi-square test for group(SHG versus non-SHG) comparison with significant p-value shown as ***: p<0.01, **: p<0.05 ,* and p<0.10. **Note:** Non-member households are those which do not include a SHG member and SHG households are those that include a SHG member.

For peer review only - http://bmjopen.bmj.com/site/about/guidelines.xhtml

Patient and public involvement

No patients or community members were directly involved in this study.

Multivariable logistic regression analysis results: Models I-IV

The results from multivariable logistic regression models are presented in Table 3 for Models I and II (effects of IMFHL program exposure and survey round), and in Table 4 for Models III and IV (effects of IMFHL program exposure and survey round adjusting for socio-demographic, economic and area-level confounders). The Odds Ratio (OR), along with their associated 95 per cent confidence intervals (95% CI) are reported. The a priori level of significance was set at the usual 5% alpha and all p-values reported in Tables using the asterisk convention: ***: $p < 0.01$; **: $p < 0.05$; *: $p < 0.10$, with the last category meant to show that a 'trend towards statistical significance' has to be noted.

Model I: IMFHL program's effects on birth preparedness and complication readiness

Women belonging to a SHG only and those belonging to and receiving the health intervention from a SHG had higher odds of reporting birth and complication readiness practice in the last pregnancy than non-SHG members in non-program (pure control) villages (Table 3). Women in SHG households (members) were 1.17 times more likely to have practised BPCR in their last pregnancy (OR=1.17, 95% CI: 1.07–1.29, p<0.01) than non-members in pure control villages. SHG members who received additional health literacy were 1.48 times more likely to practise BPCR (OR=1.48, 95% CI: 1.35–1.63, p<0.01) than non-members in pure control villages. In contrast, results also showed that non-member women living in the same villages where either SHG or SHG plus health intervention was implemented were 0.89 times *less likely* to practise BPCR compared to non-member women in pure control villages, that is, women in those villages where no SHG or health intervention was implemented (OR=0.89, 95% CI:0.82–0.97, p<0.01). Overall, results showed that women in households interviewed in round II were

For peer review only - http://bmjopen.bmj.com/site/about/guidelines.xhtml

almost 0.37 times *less likely* to have practised BPCR compared to households interviewed in round I, given all the levels of program exposure (OR=0.37, 95% CI:0.35–0.39, p<0.01).

Model II: Interaction effect of IMFHL program and survey round

Model II, table 3 showed that the inclusion of the interaction term rendered the women in SHG plus health households in round I with a statistically not significant four per cent lower odds of practising BPCR compared with non-member women in control villages in round I (OR=0.96, 95%CI: 0.84-1.11, p>0.10). As explained, the magnitude observed here corresponds to what is happening in round I (in Model II), while what was observed in Model I (1.48 times more likely to practise BPCR) corresponds to the magnitude of the exposure all rounds combined. And this is expected since there is no program exposure in Round I. The interaction of the variable survey round with household's level of microfinance exposure showed findings for each level of household IMFHL program exposure in round 2 compared to the household's same exposure level in round 1.

The results from the two-way interaction in Model II of round by households' microfinance exposure level showed that women in SHGs that received the health literacy intervention were 2.21 times more likely to practise BPCR in round II compared to the same household type in round I (OR=2.21, 95%CI: 1.82–2.68, p<0.01). This means that women in the intervention group were roughly 2.122 (=0.96*2.21) (95%CI:1.52–2.97) times more likely to practise BPCR in round II compared to women in control villages. Similarly, the results showed that non-members in program villages reported 1.73 times higher odds of BPCR practice in round II compared to the same household type in round I (OR=1.73, 95%CI: 1.46–2.05, p<0.01). These results indicate that a diffusion effect occurred from SHG women receiving health literacy to non-member women in the same villages, with women in this group being almost 1.194 (=0.69*1.73) (95%CI:0.89-1.57) times more likely to practise BPCR.

For peer review only

For peer review only - http://bmjopen.bmj.com/site/about/guidelines.xhtml

Models III and IV: Effects of IMFHL program adjusted for confounders

Table 4 shows model III and IV results where the effect of main exposure variable, IMFHL program exposure and survey round, and interaction term is assessed when confounders are added. Overall, the effects of program exposure on BPCR observed previously in model II remained unaffected when adjusting for confounders in models III and IV, suggesting that the outcome, BPCR, is mainly influenced by program exposure rather than confounders.

Key confounders: Effects of individual health and health system variables

Women who were discharged earlier from the health facility after delivery had higher odds of reporting the practice of all steps of BPCR in their last pregnancy (OR=1.15, 95%CI: 1.00–1.33, $p<0.05$). In contrast, women who reported receiving four ANC visits with key tests done in the last ANC visit reported *lower odds* of BPCR practice compared to women who received less than four visits and with incomplete tests (OR=0.90, 95%CI:0.82–0.99, $p<0.05$). Among variables reflecting the continuum of maternal care, women with the correct knowledge about the required number of ANC visits were more likely to practise BPCR compared to those with incorrect knowledge (OR=1.06, 95%CI: 0.99–1.13, $p<0.10$).

Key confounders: Effect of socio-demographic, economic and area-level variables

Model IV results for the wealth quintile variable showed that the likelihood of BPCR practice reduced as the level of household poverty increased from marginally poor to the poorest households, with the poorest households (those in the fifth wealth quintile) being 0.87 times less likely to practise BPCR than women in marginally poor households (those in the first quintile). Maternal education, which has previously been found in the literature to be strongly correlated with routine maternal health behaviours, in contrast, was negatively associated with BPCR practice in this analysis. Mothers who had completed at least primary education were

For peer review only - http://bmjopen.bmj.com/site/about/guidelines.xhtml

seven per cent less likely to practise BPCR than mothers without any schooling (OR=0.93, 95% CI: 0.87–0.99, p<0.05).

Table 3: Logistic regression models I and II results estimating levels of BPCR: Odds ratios and associated 95% confidence intervals

	Explanatory variable name	Model I	Model II
		Odds ratio (95 % CI)	Odds ratio (95 % CI)
	**Main effects**		
1.	**Level of household (HH) microfinance (MF) exposure**		
	0. HH in a village with **no program** intervention (pure control)	Reference	Reference
	1. **Non-member** HH in village with **SHG or SHG plus health intervention**	0.89*** (0.82-0.97)	0.69*** (0.61-0.77)
	2. **SHG member** HH in village with **SHG only intervention**	1.17*** (1.07-1.29)	1.13* (0.98-1.30)
	3. **SHG plus health member** HH in village with **SHG *plus* health** intervention	1.48*** (1.35-1.63)	0.96 (0.84-1.11)
2.	**Round**		
	Round 1	Reference	Reference
	Round 2	0.37*** (0.35-0.39)	0.25*** (0.22-0.29)
3.	**Two-way interaction effects: Round # households (HH) microfinance (MF) Exposure**	**Interaction term effects**	
	Round 1# Non-MF HH in pure control village		Reference
	Round 2 # Non-MF HH in a village with MF or MF plus health	----	1.73*** (1.46-2.05)
	Round2 # **SHG member** HH in village with MF program only	----	1.10 (0.90-1.33)
	Round2 # **SHG plus health member** HH in village with MF plus health intervention	----	2.21*** (1.82-2.68)

Note: Confidence intervals in parentheses; significant p-value shown as ***: p<0.01, **: p<0.05 and *: p<0.10.
BPCR: birth preparedness and complication readiness.

Table 4: Logistic regression models III and IV results estimating levels of BPCR using confounders: Odds ratios and associated 95% confidence intervals

S.no	Explanatory variable name	Model III	Model IV
		Odds ratio (95 % CI)	Odds ratio (95 % CI)
1.	**Level of household (HH) microfinance (MF) exposure** 1. HH in a village with no program intervention (Pure control)	Reference	Reference
	2. **Non-member** HH in a village with **SHG or SHG plus health intervention**	0.69*** (0.61-0.78)	0.70*** (0.62-0.78)
	3. **SHG member** HH in a village with **SHG only intervention**	1.13* (0.98-1.30)	1.14* (0.99-1.31)
	4. **SHG plus health member** HH in a village with **SHG *plus* health** intervention	0.97 (0.84-1.11)	0.98 (0.85-1.12)
2	**Round:** [Round 1]	Reference	Reference
	Round 2	0.24*** (0.21-0.28)	0.24*** (0.20-0.27)
3.	**Two-way interaction effects (round # SHG exposure)** *[Round 1# Non-MF HH in pure control village]*	Reference	Reference

For peer review only

S.no	Explanatory variable name	Model III	Model IV
	Round 2 # Non-MF HH in village with MF or MF plus health	1.72*** (1.45-2.04)	1.72*** (1.45-2.04)
	Round2 # MF-HH in village with MF only	1.10 (0.90-1.33)	1.10 (0.90-1.33)
	Round2 # MF plus health HH in village with MF plus health	2.20*** (1.82-2.67)	2.20*** (1.81-2.66)
	Model III using individual health and health system confounders		
4.	**Parity (number of previous pregnancies) of Eligible Woman (EW)**	0.98 (0.96-1.00)	0.98 (0.95-1.01)
5.	**Any past pregnancy loss (due to spontaneous/induced abortion) :** [No previous pregnancy loss]	Reference	Reference
	Previous pregnancy loss	0.98 (0.91-1.05)	0.98 (0.90-1.05)
6.	**Any complication experienced in last pregnancy/labour or postpartum:**[No complication experienced]	Reference	Reference
	Complication experienced	0.95 (0.89-1.01)	0.95 (0.89-1.01)
7.	**EW's knowledge of the minimum number of antenatal check-ups (ANC) required during pregnancy:** [Incorrect knowledge]	Reference	Reference
	Correct knowledge	1.07** (1.00-1.14)	1.06* (0.99-1.13)
8.	**Received four or more ANC in last pregnancy (with urine/blood pressure /weight/abdominal/ultrasound tested in last ANC):** [Not received]	Reference	Reference
	Received four ANC with all tests done in last ANC	0.92 (0.85-1.01)	0.91** (0.82-0.99)
9.	**Place of last delivery:** [Home delivery]	Reference	Reference
	Institutional delivery	0.95 (0.81-1.11)	0.92 (0.79-1.08)
10.	**Duration (in hours) of stay in health facility immediately after delivery**:[Home delivery]	Reference	Reference
	Discharged within 12 hrs	1.14* (0.98-1.31)	1.15** (0.99-1.33)
	Discharged between 12–24 hrs	1.09 (0.92-1.29)	1.10 (0.93-1.30)
	Discharged between 24–48 hrs	0.98 (0.82-1.17)	0.99 (0.83-1.18)
	Discharged between 48–72 hrs	1.02 (0.81-1.29)	1.05 (0.83-1.32)
	Discharged after >72 hrs	1.00	1.00
11.	**Received three postnatal care visits (PNC) in first seven days after delivery:** [Not received any PNC or received after seven days]	Reference	Reference
	Received three PNC in first seven days after delivery	1.06 (0.95-1.19)	1.05 (0.94-1.18)
12.	**Number of contacts with ASHA/ANM/AWW/SHG in last pregnancy**	0.99 (0.99-1.00)	1.00 (0.99-1.00)
13.	**Distance to primary health centre if not available in the village**	0.99 (0.98-1.00)	0.99 (0.99-1.00)
	Model IV using socio-demographic/economic and area-level confounders		
14.	**Village distance (kilometres) to closest town**	----	0.99 (1.03-1.13)
15.	**Population of village**	----	1.00 (0.99-1.00)
16.	**Household (HH) with Below Poverty Line (BPL) Card:** [No- HH does not have BPL Card]	----	Reference

For peer review only - http://bmjopen.bmj.com/site/about/guidelines.xhtml

S.no	Explanatory variable name	Model III	Model IV
	Yes – HH has BPL Card	----	1.01 (0.94-1.08)
17.	**Household wealth quintile (poor to poorest)** 1. Marginally poor	----	Reference
	2. Moderately poor	----	1.11** (1.00-1.22)
	3. Poor	----	1.00 (0.90-1.10)
	4. Poorer	----	0.91* (0.81-1.01)
	5. Poorest	----	0.87** (0.77-0.97)
18.	**EW presently working to earn cash, in-kind or both:** [Not working]	----	Reference
	Currently working	----	1.00 (0.92-1.09)
19.	**Family type:** [Nuclear]	----	Reference
	Joint and Extended	----	1.05 (0.98-1.13)
20.	**Religion:** [Muslim]	----	Reference
	Hinduism & others	----	1.06 (0.94-1.19)
21.	**Scheduled caste:**[General caste]	----	Reference
	Other backward castes	----	0.96 (0.87-1.07)
	Scheduled caste and scheduled tribe	----	0.97 (0.87-1.08)
22.	**EW age in completed years**	----	1.00 (0.99-1.01)
23.	**EW education level:**[No schooling]	----	Reference
	Completed primary/middle School (up to Year 9) and above	----	0.96 (0.89-1.03)
24.	**EW's husband education level:** [No schooling]	----	Reference
	Completed primary school/middle school (up to Year 9) and above	----	1.06 (0.97-1.17)
	**Estimation of model fit**	**Model III**	**Model IV**
	Log likelihood	-11,354	-11,334
	Number of observations	17,244	17,244
	AIC/BIC	22,750/22,914	22,741/ 23,021

Notes: Confidence intervals in parentheses; significant p-value shown as ***: p<0.01, **: p<0.05 and *: p<0.10. Log-likelihood and AIC/BIC values are also reported.

Discussion

This study makes a significant contribution to the literature on maternal health promotion and implementation by investigating the main effect of IMFHL program membership on birth preparedness and complication readiness (BPCR) practice among poor women who have recently given birth in rural UP. The study provides evidence of the impacts of SHG membership alone and when integrated with health literacy on BPCR practice. This evidence

For peer review only

For peer review only - http://bmjopen.bmj.com/site/about/guidelines.xhtml

suggests that BPCR practice is potentially improved during maternal complications among SHG members when SHGs were integrated with the health literacy program and supports the claim that microfinance-based women's groups are an effective strategy to provide health literacy to marginalised rural populations enabling behaviour change. No previous study has examined the impact on BPCR in the context of SHG programs alone or in an integrated SHG and health literacy intervention program in India or elsewhere. This is also the first study to find the presence of a diffusion effect of maternal health promotion behaviour — BPCR practice—from SHG members receiving health literacy to non-members in program villages.

Overall, the results reveal that SHGs promote the adoption of BPCR practice among members and the odds of BPCR adoption almost doubles when a health literacy component is integrated into the model. However, the diffusion effect of BPCR practice is only observed when SHGs have an integrated health literacy component. The study finds the diffusion of BPCR practices among non-members in SHG plus health literacy villages and not in the SHG-only villages.

Therefore, these findings suggest that self-help groups require the addition of a maternal health literacy component to achieve a higher coverage of a behavioural health intervention among members and non-members in villages. The underlying mechanism of diffusion requires additional research to explain pathways; however, insights from previous studies suggest that either SHG members may themselves be promoting practices as 'model adopters' of new behaviour through interpersonal contact, or that SHGs serve as a better platform for facilitating linkages between non-members and community health workers when combined with health literacy intervention (36,37,39). It is, therefore, feasible that when SHG members receive health literacy, they may be acting as change agents to encourage non-members to adopt desired health behaviours. The study further highlighted that complex health behaviour such as BPCR, which comprises multiple steps to constitute full practice, in the absence of program intervention,

For peer review only - http://bmjopen.bmj.com/site/about/guidelines.xhtml

396 declined with time reflecting an overall negative secular effect of time. The IMFHL program
397 thus has a protective effect of pulling up these lagging indicators, which are otherwise likely to
398 keep decreasing and negatively impact maternal health.

399 This study highlights the important role that SHGs play as a catalyst for promoting maternal
400 health behavioural change when layered up with health literacy intervention. The findings are
401 supported by other studies from UP and Bihar, another Indian state with low development
402 indicators, that had shown an increase in routine indicators of maternal health system utilisation
403 (such as four ANC visits and institutional delivery) and home-based neonatal health care
404 practices by 5 to 11 percentage points when health literacy intervention was provided through
405 SHGs compared to membership in SHGs alone (33,40). In this study, the higher likelihood of
406 BPCR practise among women in SHGs plus health intervention households could be a direct
407 consequence of SHG membership that provides a platform, which gives women access to
408 needed funds. This access addresses one of the key BPCR steps—saving funds for a maternal
409 emergency that influences care-seeking behaviours in low-income families. High maternal
410 health expenses are seen as a strong factor for pushing vulnerable families at risk of poverty
411 into debt traps that reinforce debt, borrowing, and illness cycles. Therefore, in such settings, the
412 provision of health literacy and financial access together are expected to improve households'
413 care-seeking preferences. Previous studies have also shown that SHGs provide mutual support
414 among members (33,36). Consequently, health literacy delivered through the peer network of
415 SHGs is likely to change health practices among marginalised women.

416 In adjusting for other socioeconomic confounders, this study also found that across wealth
417 quintiles, a significant negative effect in BPCR practices was observed only in the poorest (fifth
418 quintile) households, with these households least likely to practise BPCR.

For peer review only

For peer review only - http://bmjopen.bmj.com/site/about/guidelines.xhtml

Study strengths and limitations

This was a first-of-its-kind study in rural India that comprehensively evaluated the impact of an integrated microfinance and health literacy program on uptake of BPCR practice which can reduce delays in maternal care-seeking during complications.

A key strength of the study relates to the large sample size of 17244 women representing marginalised rural populations compared to other studies. Additionally, the study adds value to community evaluation research by using a quasi-experimental study design with pre-and post-intervention measurements, which is potentially the efficient approach in a highly populated developing country setting, providing the opportunity to evaluate the incremental change across program exposure levels (impact of SHG only, SHG plus health intervention), and the potential diffusion effect (impact of SHG plus health intervention from members to non-members in areas without any program exposure).

Investigating the diffusion effect of practice from groups who received the intervention to neighbouring non-member households generated evidence on the expanded reach of such an intervention, leading to greater coverage of vulnerable populations. The key findings, however, should be interpreted while considering the following study limitations.

First, the selection of program blocks under the IMFHL was based on the program's operational criterion, thus preventing randomisation. The quasi-experimental survey design, however, adopted a multistage sampling approach using different criteria to select blocks (SC/ST percentage) and villages (SHG coverage and population size) to limit selection bias and allow for comparisons of areas with similar socio-cultural and economic characteristics.

For peer review only - http://bmjopen.bmj.com/site/about/guidelines.xhtml

Also, the survey collected information from women based on self-recall, which may suffer from recall bias and social desirability. However, the potential for these errors is minimised as the women were interviewed within 12 months post-delivery and by trained interviewers.

The IMFHL program's definition of SHG membership which consisted of the pregnant woman herself or her mother-in-law or sister in law is an important consideration for understanding how the health literacy component informed the pregnant woman's health behaviour change in intervention areas. In intervention areas, the pregnant woman received health information directly from the SHG whether she was a member or lived in a household where the mother in law or sister in law were members. The pregnant woman was invited to attend health literacy meetings in the SHG. In the program, the majority(2/3rds) of the pregnant woman was SHG members themselves, and (1/3rd) lived in a household where the mother in law or sister in law was the member. Thus, for some pregnant women, the SHG platform may have a broader role in involving other family members in the health literacy discussions. This is an important consideration in rural UP where family members, especially the mother-in-law, influence the adoption of maternal health behaviours.

Finally, while the survey design selected women from SHG member and non-member households from the same villages, in some cases, the women sampled in a given village would be either all members or all non-members, which potentially limited the variation in the outcome variable within villages.

Policy and program implications

A programmatic strength of the IMFHL intervention was that it utilised the existing structure of the SHG program to embed the health literacy component, which provided evidence of a scalable model for disseminating health information. The findings reported in this study are

For peer review only - http://bmjopen.bmj.com/site/about/guidelines.xhtml

relevant for the rural Indian context, where the Indian Government is establishing a network of women-only SHGs as a national poverty alleviation program. Therefore, allowing the integration of the health intervention into SHG structures, as the IMFHL program created the opportunity to improve maternal health outcomes for the state and the country.

Lastly, as healthcare-seeking behaviours are shaped by cultural practices in community settings, the diffusion effect of health messages from members to non-members can reinforce desired health behaviours. Diffusion also increases program coverage of harder to reach populations. Future research is required to explore the pathways of the diffusion effect of knowledge and practice from members to non-members in program villages.

Conclusions

The integrated microfinance and health literacy (IMFHL) program evaluated in this study promoted behaviour changes amongst some of the most marginalised households in UP, India's most populous state and one of its most developmentally disadvantaged areas. The findings show that SHGs exert both a dissemination effect of planned health behaviour within members as well as a diffusion effect of the natural transfer of BPCR practice from members to non-members but only when a health literacy component is added to SHGs. The findings from this research support integrating health literacy components into SHG networks and similar community groups to promote households' adoption of complication readiness plans to reduce delays in seeking health care during maternal complications.

Lessons from the IMFHL program implementation in India provide the opportunity to adapt SHG models implemented elsewhere by adding health literacy components. Beyond India, microfinance is promoted globally as a poverty alleviation program in low resource settings where the burden of maternal mortality remains high. These types of interventions' can assist

For peer review only - http://bmjopen.bmj.com/site/about/guidelines.xhtml

For peer review only

other low resource settings in accelerating progress towards the maternal health target of the Sustainable Development Goals.

References

1. Berman P, Requejo J, Bhutta ZA, Singh NS, Owen H, Lawn JE. Countries' progress for women's and children's health in the Millennium Development Goal era: the Countdown to 2015 experience. BMC Public Health [Internet]. 2016;16(S2):791. Available from: http://bmcpublichealth.biomedcentral.com/articles/10.1186/s12889-016-3398-x

2. Moucheraud C, Owen H, Singh NS, Ng CK, Requejo J, Lawn JE, et al. Countdown to 2015 country case studies: what have we learned about processes and progress towards MDGs 4 and 5? BMC Public Health [Internet]. 2016 [cited 2017 Oct 19];16(2). https://doi.org/10.1186/s12889-016-3401-6

3. United Nations. The Millennium Development Goals Report. United Nations. 2015;72.

4. Alkema L, Chou D, Hogan D, Zhang S, Moller AB, Gemmill A, et al. Global, regional, and national levels and trends in maternal mortality between 1990 and 2015, with scenario-based projections to 2030: A systematic analysis by the un Maternal Mortality Estimation Inter-Agency Group. Lancet [Internet]. 2016;387(10017):462–74. Available from: http://dx.doi.org/10.1016/S0140-6736(15)00120-8

5. Department of Economic and Social Affairs, United Nations. The Sustainable Development Goals Report [Internet]. United Nations. New York; 2016. Available

For peer review only - http://bmjopen.bmj.com/site/about/guidelines.xhtml

from: https://unstats.un.org/sdgs/report/2019/The-Sustainable-Development-Goals-Report-2019.pdf

6. Trends in maternal mortality 2000 to 2017: estimates by WHO, UNICEF, UNFPA, World Bank Group and the United Nations Population Division. [Internet]. World Health Organization. Geneva; 2019. Available from: https://www.who.int/reproductivehealth/publications/maternal-mortality-2000-2017/en/

7. Ronsmans, C., & Graham W. Maternal mortality : who , when , where , and why. Lancet [Internet]. 2006;368(9542):1189–200. Available from: https://www.thelancet.com/journals/lancet/article/PIIS014067360669380X/fulltext

8. Gowda SD. Millennium Development Goals - Final Country Report of India. 2017;(September):222. Available from: http://www.mospi.gov.in/sites/default/files/publication_reports/MDG_Final_Country_report_of_India_27nov17.pdf%0Awww.mospi.gov.in

9. Kassebaum NJ, Barber RM, Bhutta ZA, Dandona L, Gething PW, Hay SI, et al. Global, regional, and national levels of maternal mortality, 1990–2015: a systematic analysis for the Global Burden of Disease Study 2015. Lancet [Internet]. 2016;388(10053):1775–812. Available from: http://linkinghub.elsevier.com/retrieve/pii/S0140673616314702

10. Office of The Registrar General of India. Special Bulletin on Maternal Mortality in India 2015-17 [Internet]. 2019. Available from: http://www.censusindia.gov.in/vital_statistics/SRS_Bulletins/MMR_Bulletin-2015-17.pdf

For peer review only - http://bmjopen.bmj.com/site/about/guidelines.xhtml

11. Koblinsky M, Moyer CA, Calvert C, Campbell J, Campbell OMR, Feigl AB, et al. Quality maternity care for every woman, everywhere: a call to action. Lancet [Internet]. 2016;388(10057):2307–20. Available from: http://dx.doi.org/10.1016/S0140-6736(16)31333-2

12. Graham W, Woodd S, Byass P, Filippi V, Gon G, Virgo S, et al. Diversity and divergence: the dynamic burden of poor maternal health. Lancet [Internet]. 2016;388(10056):2164–75. Available from: http://dx.doi.org/10.1016/S0140-6736(16)31533-1

13. Countdown to 2015. Maternal, New Born and Child Survival: India 2015 report. 2015;(000):142–3.

14. Mission of Health and Family Welfare. 10th Common review mission [Internet]. 2016. Available from: https://nhm.gov.in/index1.php?lang=1&level=2&sublinkid=825&lid=448

15. Saxena D, Vangani R, Mavalankar D V., Thomsen S. Inequity in maternal health care service utilisation in Gujarat: Analyses of district-level health survey data. Glob Health Action. 2013;6(1). doi:10.3402/gha.v6i0.19652

16. Knight HE, Self A, Kennedy SH. Why Are Women Dying When They Reach Hospital on Time? A Systematic Review of the "Third Delay." PLoS One. 2013;8(5). doi: 10.1371/journal.pone.0063846

17. Sri S, Khanna R, Abhiyan JS. Dead Women Talking [Internet]. Dead Women Talking. 2018 [cited 2017 Nov 9]. Available from: http://www.commonhealth.in/Dead Women

For peer review only - http://bmjopen.bmj.com/site/about/guidelines.xhtml

Talking full report final.pdf

18. Gabrysch S, Campbell OMR. Still too far to walk: Literature review of the determinants of delivery service use. BMC Pregnancy Childbirth [Internet]. 2009;9(1):34. Available from: https://doi.org/10.1186/1471-2393-9-34

19. Chaturvedi S, Randive B, Diwan V, De Costa A. Quality of obstetric referral services in India's JSY cash transfer programme for institutional births: A study from madhya pradesh province. PLoS One. 2014;9(5). https://doi.org/10.1371/journal.pone.0096773

20. Institute for Health Metrics and Evaluation (IHME). GBD PROFILE : INDIA. Global Burden of Disease Study 2010 (GBD 2010). 2010;2010(Gbd):1–4. Available from: http://www.healthdata.org/sites/default/files/files/country_profiles/GBD/ihme_gbd_country_report_india.pdf.

21. United Nations. India and the MDGs Towards a sustainable future for all. 2015.

22. Sri S, Khanna R, Abhiyan JS. Dead Women Talking A civil society report on maternal deaths in India People's Health Movement -India. [cited 2017 Nov 12]; Available from: http://www.commonhealth.in/Dead Women Talking full report final.pdf [Internet]. 2004 [cited 2017 Dec 2];364(11). https://doi.org/10.1016/S0140-6736(04)17021-9

23. Mbalinda SN, Nakimuli A, Kakaire O, Osinde MO, Kakande N, Kaye DK. Does knowledge of danger signs of pregnancy predict birth preparedness? A critique of the evidence from women admitted with pregnancy complications. Health Research and Policy Systems. 2014;12(1):1–7.

For peer review only

For peer review only - http://bmjopen.bmj.com/site/about/guidelines.xhtml

24. Campbell O, Graham WJ. Maternal Survival 2 Strategies for reducing maternal mortality : getting on with what works. 2006;368:1284–99. Available from: https://pubmed.ncbi.nlm.nih.gov/17027735/

25. Markos, Desalegn Bogale D. Birth preparedness and complication readiness among women of child bearing age group in Goba woreda, Oromia region, Ethiopia. BMC Pregnancy Childbirth [Internet]. 2014;14(1):1–9. Available from: http://www.embase.com/search/results?subaction=viewrecord&from=export&id=L600014040%5Cnhttp://dx.doi.org/10.1186/1471-2393-14-282

26. Mbachu II, Ezeama C, Osuagwu K, Umeononihu OS, Obiannika C, Ezeama N. A cross sectional study of maternal near miss and mortality at a rural tertiary centre in southern nigeria. BMC Pregnancy Childbirth. 2017;17(1):1–8.

27. Kananura RM, Tetui M, Bua J, Ekirapa-Kiracho E, Mutebi A, Namazzi G, et al. effect of a participatory multisectoral maternal and newborn intervention on birth preparedness and knowledge of maternal and newborn danger signs among women in Eastern Uganda: A quasi-experiment study. Glob Health Action [Internet]. 2017;10(0). Available from: https://doi.org/10.1080/16549716.2017.1362826

28. Baqui AH, Williams EK, Rosecrans AM, Agrawal PK, Ahmed S, Darmstadt GL, et al. impact of an integrated nutrition and health programme on neonatal mortality in rural northern India. Bull World Health Organ. 2008;86(10):796–804.

29 Kumar V, Mohanty S, Kumar A, Misra RP, Santosham M, Awasthi S, et al. Effect of community-based behaviour change management on neonatal mortality in Shivgarh, Uttar Pradesh, India: a cluster-randomised controlled trial. Lancet.

For peer review only - http://bmjopen.bmj.com/site/about/guidelines.xhtml

2008;372(9644):1151–62.

30. Prost A, Colbourn T, Seward N, Azad K, Coomarasamy A, Copas A, et al. Women's groups practising participatory learning and action to improve maternal and newborn health in low-resource settings: A systematic review and meta-analysis. Lancet [Internet]. 2013;381(9879):1736–46. Available from: http://dx.doi.org/10.1016/S0140-6736(13)60685-6

31. Gage AJ. Barriers to the utilisation of maternal health care in rural Mali. Soc Sci Med. 2007;65(8):1666–82. https://doi.org/10.1016/j.socscimed.2007.06.001

32. Ahmad D, Mohanty I, Hazra A, Niyonsenga T. The Knowledge of Danger Signs of Obstetric Complications Among Women in Rural India : Evaluating an Integrated Microfinance and Health Literacy Program. BMC Pregnancy Childbirth 21, 79 (2021). https://doi.org/10.1186/s12884-021-03563-

33. Hazra A, Atmavilas Y, Hay K, Saggurti N, Verma RK, Ahmad J, et al. Effects of health behaviour change intervention through women's self-help groups on maternal and newborn health practices and related inequalities in rural india: A quasi-experimental study. EClinicalMedicine [Internet]. 2020;18:100198. Available from: https://doi.org/10.1016/j.eclinm.2019.10.011

34. Singh R, Neogi SB, Hazra A, Irani L, Ruducha J, Ahmad D, Kumar S, Mann N, Mavalankar D. Utilization of maternal health services and its determinants: a cross-sectional study among women in rural Uttar Pradesh, India. J Heal Popul Nutr [Internet]. 2019;38(13):1–12. Available from: http://doi.org/10.1186/s41043-019-0173-5

For peer review only

For peer review only - http://bmjopen.bmj.com/site/about/guidelines.xhtml

35. Lyngdoh T, Neogi SB, Ahmad D, Soundararajan S, Mavalankar D. Intensity of contact with frontline workers and its influence on maternal and newborn health behaviors: cross-sectional survey in rural Uttar Pradesh, India. J Health Popul Nutr. 2018;37(1):2. Published 2018 Jan 8. doi:10.1186/s41043-017-0129-6

36. Ruducha J, Hariharan D, Potter J, et al.Measuring coordination between women's self-help groups and local health systems in rural India: a social network analysis.BMJ Open 2019;9:e028943. doi: 10.1136/bmjopen-2019-028943

37. Ruducha J, Huang X, Potter J, Hariharan D, Ahmad D, Kumar S, et al. Perceived Social Networks and Newborn Health: Evidence from Self-Help Group Communities in Northern India. Societies [Internet]. 2018;8(4):92. Available from: http://doi.org/10.3390/soc8040092

38. Census of India : Provisional Population Totals Paper 1 of 2011 : Uttar Pradesh [Internet]. [cited 2019 Nov 30]. Available from: http://www.censusindia.gov.in/2011-prov-results/prov_data_products_up.html

39. Devasenapathy N, Neogi SB, Soundararajan S, Ahmad D, Hazra A, Ahmad J, et al. Association of antenatal care and place of delivery with newborn care practices: evidence from a cross-sectional survey in rural Uttar Pradesh, India. J Heal Popul Nutr [Internet]. 2017;36(1):30. Available from: http://doi.org/10.1186/s41043-017-0107-z

40. Walia M, Irani L, Chaudhuri I, Atmavilas Y, Saggurti N. Effect of sharing health messages on antenatal care behavior among women involved in microfinance-based self-help groups in Bihar India. Glob Heal Res Policy. 2020;5(1):1–8

For peer review only - http://bmjopen.bmj.com/site/about/guidelines.xhtml

Declarations

Ethics Statement

Ethics approval was granted by the Human Research Ethics Committee, at the University of Canberra for the program '*2266 - PhD:The Impact of An Integrated Health Literacy and Microfinance Program on Care Awareness and Care Seeking Behaviour During Maternal Complication: The Case of Rural India'*. Previously, an independent ethics committee of Population Council, New York, USA, approved the primary data collection under the IMFHL program.

Role of Funders.

The funders had no role in study design, data collection and analysis, decision to publish, or preparation of the manuscript.

Acknowledgments

We wish to thank Professor Rachel Davey, Health Research Institute, the University of Canberra, for her guidance and for reviewing the final draft of this manuscript. The authors would also like to thank the University of Canberra (Australia), the Public Health Foundation of India (India) and the Population Council (India) for supporting this international research collaboration.

Competing Interests

DA, IM and TN have no competing interests.

Funding

The paper was part of the PhD research of the first author (Danish Ahmad), who was supported by the University of Canberra's Higher Degree by Research scholarship for his

For peer review only - http://bmjopen.bmj.com/site/about/guidelines.xhtml

PhD. The Bill and Melinda Gates Foundation provides open access funding for the publication; they also funded the IMFHL program evaluated in the paper.

Authors Contributions

DA conceptualised the study, conducted the analysis, interpreted the results and drafted the study. IM guided the study design, the data analysis, the interpretation of the results and discussion. TN guided the study design, the data analysis and interpretation of results and discussion. All authors read and approved the final manuscript.

Availability of data and materials

The datasets used and/or analysed during the current study are available from the corresponding author on reasonable request.

Acknowledgments

We wish to thank Professor Rachel Davey, Health Research Institute, the University of Canberra, for her guidance and for reviewing the final draft of this manuscript. The authors would also like to thank the University of Canberra (Australia), the Public Health Foundation of India (India) and the Population Council (India) for supporting this international research collaboration.

Figure legend

Figure 1: Assumed pathway of knowledge diffusion from member households (tier I) to neighbouring non-member households (tier II) within villages

For peer review only - http://bmjopen.bmj.com/site/about/guidelines.xhtml

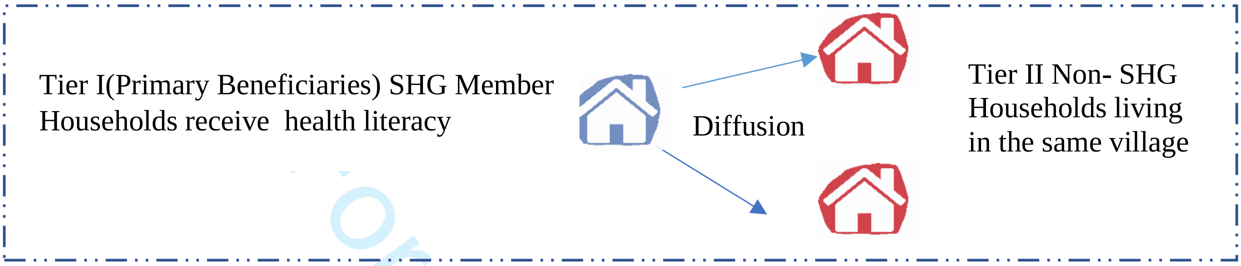

For peer review only

STROBE Statement—Checklist of items that should be included in reports of ***case-control studies***

	Item No	Recommendation	Page No
**Title and abstract**	1	(*a*) Indicate the study's design with a commonly used term in the title or the abstract	2
		(*b*) Provide in the abstract an informative and balanced summary of what was done and what was found	2
**Introduction**			
Background/rationale	2	Explain the scientific background and rationale for the investigation being reported	3-6
Objectives	3	State-specific objectives, including any prespecified hypotheses	6
**Methods**			
Study design	4	Present key elements of study design early in the paper	8-9
Setting	5	Describe the setting, locations, and relevant dates, including periods of recruitment, exposure, follow-up, and data collection	8
Participants	6	(*a*) Give the eligibility criteria and the sources and methods of case ascertainment and control selection. Give the rationale for the choice of cases and controls	7
		(*b*) For matched studies, give matching criteria and the number of controls per case	8(references 32,33)
Variables	7	Clearly define all outcomes, exposures, predictors, potential confounders, and effect modifiers. Give diagnostic criteria, if applicable	9-11
Data sources/ measurement	8*	For each variable of interest, give sources of data and details of methods of assessment (measurement). Describe comparability of assessment methods if there is more than one group	6,10
Bias	9	Describe any efforts to address potential sources of bias	23-24
Study size	10	Explain how the study size was arrived at	8 -9(lines 188-191
Quantitative variables	11	Explain how quantitative variables were handled in the analyses. If applicable, describe which groupings were chosen and why	9-11
Statistical methods	12	(*a*) Describe all statistical methods, including those used to control for confounding	10-11
		(*b*) Describe any methods used to examine subgroups and interactions	9-10,16,17
		(*c*) Explain how missing data were addressed	na
		(*d*) If applicable, explain how matching of cases and controls was addressed	8-9
		(*e*) Describe any sensitivity analyses	na
**Results**			
Participants	13*	(a) Report numbers of individuals at each stage of study—eg numbers potentially eligible, examined for eligibility, confirmed eligible, included in the study, completing follow-up, and analysed	8,9
		(b) Give reasons for non-participation at each stage	8
		(c) Consider use of a flow diagram	Ref 32 on page 8
Descriptive data	14*	(a) Give characteristics of study participants (eg demographic, clinical, social) and information on exposures and potential confounders	8

For peer review only - http://bmjopen.bmj.com/site/about/guidelines.xhtml

		(b) Indicate number of participants with missing data for each variable of interest	8
Outcome data	15*	Report numbers in each exposure category, or summary measures of exposure	13
Main results	16	(*a*) Give unadjusted estimates and, if applicable, confounder-adjusted estimates and their precision (eg, 95% confidence interval). Make clear which confounders were adjusted for and why they were included	20
		(*b*) Report category boundaries when continuous variables were categorized	20
		(*c*) If relevant, consider translating estimates of relative risk into absolute risk for a meaningful time period	na
Other analyses	17	Report other analyses done—eg analyses of subgroups and interactions, and sensitivity analyses	17-18
**Discussion**			
Key results	18	Summarise key results with reference to study objectives	11-12,16-17
Limitations	19	Discuss limitations of the study, taking into account sources of potential bias or imprecision. Discuss both direction and magnitude of any potential bias	23-25
Interpretation	20	Give a cautious overall interpretation of results considering objectives, limitations, multiplicity of analyses, results from similar studies, and other relevant evidence	20-25
Generalisability	21	Discuss the generalisability (external validity) of the study results	24-25
**Other information**			
Funding	22	Give the source of funding and the role of the funders for the present study and, if applicable, for the original study on which the present article is based	33

*Give information separately for cases and controls.

Note: An Explanation and Elaboration article discusses each checklist item and gives methodological background and published examples of transparent reporting. The STROBE checklist is best used in conjunction with this article (freely available on the Web sites of PLoS Medicine at http://www.plosmedicine.org/, Annals of Internal Medicine at http://www.annals.org/, and Epidemiology at http://www.epidem.com/). Information on the STROBE Initiative is available at http://www.strobe-statement.org.

For peer review only
